# Episodes of fissure formation in the Alps: connecting quartz fluid inclusion, fissure monazite age, and fissure orientation data

**DOI:** 10.1186/s00015-021-00391-9

**Published:** 2021-05-10

**Authors:** Edwin Gnos, Josef Mullis, Emmanuelle Ricchi, Christian A. Bergemann, Emilie Janots, Alfons Berger

**Affiliations:** 1 Natural History Museum of Geneva, Route de Malagnou 1, 1208 Geneva, Switzerland; 2 Department of Earth Sciences, University of Basel, Bernoullistrasse 32, 4056 Basel, Switzerland; 3 Department of Earth Sciences, University of Geneva, Rue de Maraîchers 13, 1205 Geneva, Switzerland; 4 Institute of Geosciences, Heidelberg University, Im Neuenheimer Feld 236, 69120 Heidelberg, Germany; 5 University of Grenoble, ISTerre, 38041 Grenoble, France; 6 Insitute of Geological Sciences, University of Bern, Baltzerstrasse 8+10, 3012 Bern, Switzerland

**Keywords:** Fluid inclusions, Fissure monazite age, Alps, Quartz habit, Tectonic evolution, Stress field

## Abstract

Fluid assisted Alpine fissure-vein and cleft formation starts at prograde, peak or retrograde metamorphic conditions of 450–550 °C and 0.3–0.6 GPa and below, commonly at conditions of ductile to brittle rock deformation. Early-formed fissures become overprinted by subsequent deformation, locally leading to a reorientation. Deformation that follows fissure formation initiates a cycle of dissolution, dissolution/reprecipitation or new growth of fissure minerals enclosing fluid inclusions. Although fissures in upper greenschist and amphibolite facies rocks predominantly form under retrograde metamorphic conditions, this work confirms that the carbon dioxide fluid zone correlates with regions of highest grade Alpine metamorphism, suggesting carbon dioxide production by prograde devolatilization reactions and rock-buffering of the fissure-filling fluid. For this reason, fluid composition zones systematically change in metamorphosed and exhumed nappe stacks from diagenetic to amphibolite facies metamorphic rocks from saline fluids dominated by higher hydrocarbons, methane, water and carbon dioxide. Open fissures are in most cases oriented roughly perpendicular to the foliation and lineation of the host rock. The type of fluid constrains the habit of the very frequently crystallizing quartz crystals. Open fissures also form in association with more localized strike-slip faults and are oriented perpendicular to the faults. The combination of fissure orientation, fissure quartz fluid inclusion and fissure monazite-(Ce) (hereafter monazite) Th–Pb ages shows that fissure formation occurred episodically (1) during the Cretaceous (eo-Alpine) deformation cycle in association with exhumation of the Austroalpine Koralpe-Saualpe region (~ 90 Ma) and subsequent extensional movements in association with the formation of the Gosau basins (~ 90–70 Ma), (2) during rapid exhumation of high-pressure overprinted Briançonnais and Piemontais units (36–30 Ma), (3) during unroofing of the Tauern and Lepontine metamorphic domes, during emplacement and reverse faulting of the external Massifs (25–12 Ma; except Argentera) and due to local dextral strike-slip faulting in association with the opening of the Ligurian sea, and (4) during the development of a young, widespread network of ductile to brittle strike-slip faults (12–5 Ma).

**Supplementary Information:**

The online version contains supplementary material available at 10.1186/s00015-021-00391-9.

## Introduction

Fluid-assisted fissure-vein and cleft formation occurred in the Alps in metamorphic rocks due to fluid-assisted embrittlement under prograde, peak to retrograde metamorphic conditions at or below 450–550 °C and 0.3–0.6 GPa (e.g., Diamond & Tarantola, 2015; Heijboer et al., 2003a, 2006; Mullis & Tarantola, 2015; Mullis, 1974, 1976a, 1976b, 1983, 1991, 1996, 2011; Mullis et al., 1994; Poty, 1969; Poty et al., 2007, 2018; Rauchenstein-Martinek et al., 2016; Sharp et al., 2005). The formation of these structures was related to the prevailing local stress fields induced by the collision of continental microplates with the European continental plate (e.g. Handy et al., 2010). Generated fissures are most commonly oriented perpendicular to foliation and lineation of their host rocks. Interaction of fluid-filled clefts with the surrounding rock led to dissolution of minerals in the wall rock and mineral precipitation in the fissures (e.g., Heijboer et al., 2006; Mullis, 1976a, 1995, 2011; Mullis & De Capitani, 2000; Mullis & Wolf, 2013; Mullis et al., 1994; Sharp et al., 2005; Weisenberger & Bucher, 2011). Fissures became either completely filled and became mineral veins, or were enlarged by subsequent tectonic activity to form fluid-filled dm- to m-sized clefts, in which large crystals grew from cleft formation down to temperatures < 200 °C. As long as deformation continued, minerals in fluid-filled clefts reacted to deformation via dissolution–precipitation cycles due to disequilibrium between fluid, rock wall and mineral assemblage within the cleft (Bergemann et al., 2019, 2020; Heijboer et al., 2003a, 2006; Mullis, 1976a, 2011; Ricchi et al., 2019, 2020a; Wolf & Mullis, 2012). Thus, minerals did not only grow following the initial fissure formation but continued to grow, to crystallize newly or to dissolve during subsequent deformation stages or other causes leading to chemical disequilibrium. Fissures forming in schists during prograde metamorphism became usually deformed until they formed foliation-parallel (e.g., Miron et al., 2013), more or less boudinaged veins. However, these veins provided important competence contrast, acting as an incipient point for retrograde formation of open fissures.

In contrast to the surrounding country rock and veins, fluid-filled fissures and clefts remained highly reactive at low temperature due to the presence of fluid, permitting registration of deformation steps through mineral growth or recrystallization (e.g., Bergemann et al., 2018, 2019, 2020; Berger et al., 2013; Mullis, 1976a, 1976b, 2011; Mullis & Wolf, 2013; Mullis et al., 2001a, 2001b; Ricchi et al., 2019, 2020a).

**Fig. 1:**
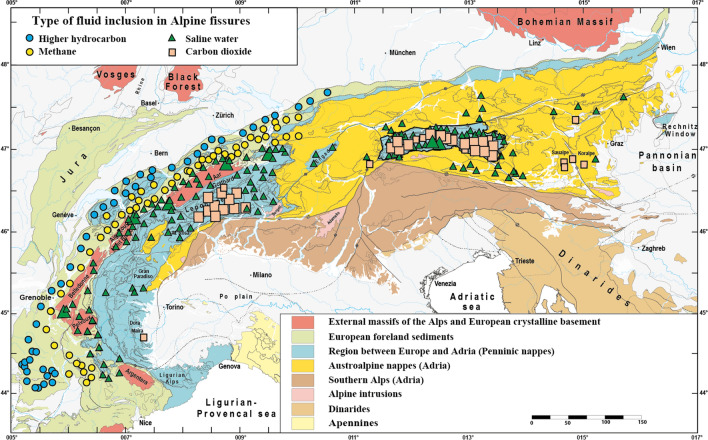
Simplified tectonic map of the Alps based on Schmid et. al. (2004) and Bousquet et. al. (2012b), showing the distribution of the earliest fluid inclusion compositions in fissure quartz. The fluid inclusion compilation in Poty et. al. (2007) was completed with more recent data. The fluid inclusion compilation is based on numerous data (Barlier, 1974; Bergemann et al., 2017, 2018; Bernard, 1978; Cesare et al., 2001; Diamond & Tarantola, 2015; Felix & Mullis, 2013; Frey et al., 1980a, 1980b; Hasenberger, 1996; Heijboer et al., 2003a, 2003b; Hetherington et al. 2003; Jourdan et al., 2009; Kandutsch, 1989; Kandutsch & Wachtler, 2000; Kandutsch et al., 1998; Klemm et al. 2004; Luckscheiter & Morteani, 1980; Martin et al., 1982; Miron et al., 2013; Mullis, 1975, 1976a, 1976b, 1979, 1983, 1987, 1988, 1996, 2011; Mullis & De Capitani, 2000; Mullis & Ramseyer, 1996; Mullis & Tarantola, 2015; Mullis et al., 1994, 2001a, 2001b, 2002, 2003; Mullis Ferreiro-Mählmann et al., 2017; Niedermayr et al., 1984; Poty, 1969; Poty & Stalder, 1970; Poty et al., 1974; Proce et al., 2015; Rahn et al., 1995; Ramseyer & Mullis, 1990; Rauchenstein-Martinek, 2014; Rauchenstein-Martinek et al., 2016; Schmidt et al., 1997; Stalder & Touray, 1970; Stalder et al., 1980; Soom, 1986; Tarantola et al., 2007, 2009; Touray et al., 1970; Wachtler & Kandutsch, 2000; Wagner et al., 1972; Wolf & Mullis, 2012; Wolf et al., 2015; Zerlauth et al., 2015). Note the distribution of the fluid types and clustering of CO_2_-dominated fluids in the Lepontine and Tauern metamorphic domes, as well as in the Koralpe-Saualpe region east of the Tauern Window. Larger symbols indicate a higher frequency of the fluid inclusion type. The Gotthard Nappe belonging to the Penninic units shows an exhumation and fissure formation history comparable to the adjacent Aar Massif (Ricchi et al., 2019)

Fissure quartz fluid inclusions similarly revealed growth events related to tectonic activity and have been studied systematically in the Alps (Bernard, 1978; Cesare et al., 2001; Fabre et al., 2002; Heijboer et al., 2003a, 2003b, 2006; Kandutsch et al., 1998; Marshall et al., 1998a, 1998b; Miron et al., 2013; Mullis, 1975, 1976a, 1976b, 1983, 1991, 1995, 1996, 2011; Mullis et al., 1994, 2002, 2017; Poty, 1969; Rauchenstein-Martinek et al., 2016; Rossi & Rolland, 2014; Tarantola et al., 2007, 2009). A compilation of the dominant fissure quartz fluid inclusion type compiled in Poty et. al. (2007) and complemented with additional data is shown in Fig. 1.

Fissure minerals are typically mm to cm in size, and easy to separate for further geochronological studies. This is the reason why potassium feldspars and micas from different localities were among the first minerals used for trying to date Alpine metamorphism (Purdy & Stalder, 1973). However, in many cases the authors found that the radiometric system of the fissure mineral was disturbed. For this reason, only a few meaningful fissure mineral ages existed (Köppel & Grünenfelder, 1975; Peretti et al., 1981; Purdy & Stalder, 1973; Rauchenstein-Martinek, 2014; Rolland et al., 2008; Sharp et al., 2005) before fissure monazite dating.

Fissure monazite most commonly crystallizes in Ca-poor lithologies (meta-pelites, meta-arenites, meta-granitoids). Once formed, diffusion in monazite is negligible under prevailing P–T conditions (Cherniak et al., 2004; Gardés et al., 2006, 2007) and hydrothermal monazite hence dates crystallization. Chemically and isotopically homogeneous crystals indicate a single, rapid growth episode (e.g., Grand’Homme et al., 2016a). Though crystals showing different growth domains indicative of successive growth episodes are most common. However, further hydrothermal processes can affect monazite after its initial crystallization (e.g., Janots et al., 2012). Alteration and dissolution-reprecipitation can lead to resetting of the monazite Th–U decay systems in parts or the entire crystal (e.g., Grand’Homme et al., 2016b; Seydoux-Guillaume et al., 2012). In other cases, parts or entire grains display a patchy zoning due to dissolution-reprecipitation processes (e.g., Ayers et al., 1999; Bergemann et al., 2019, 2020; Gnos et al., 2015; Grand’Homme et al., 2016b; Ricchi et al., 2019, 2020a), which is especially frequent in the Lepontine and Tauern metamorphic domes. The replacement process is associated with element fractionation resulting in crystal zones with often distinct U/Th values (Bergemann et al., 2017; Grand’Homme et al., 2016a, 2016b; Seydoux-Guillaume et al., 2012).

Although thermochronological data exist now for most parts of the Alps, constraining its exhumation history (e.g., Bertrand et al., 2015; Fox et al., 2016; Rosenberg & Berger, 2009; Vernon et al., 2008), it has been shown that Th–Pb fissure monazite domain ages, in some cases in combination with fluid inclusion data, are linkable to tectonic events (e.g., Bergemann et al., 2017, 2018, 2019, 2020; Berger et al., 2013; Janots et al., 2019; Ricchi et al., 2019, 2020a, 2020b). In some cases deformation is associated with fluid advection (e.g., Bergemann et al., 2019; Janots et al., 2019). On the other hand, since cleft monazite starts to crystallize typically ≤ 400 °C (much below the monazite closure temperature (Cherniak et al., 2004), it can be utilized to constrain successive deformation activity in fault and damage zones under low grade metamorphic conditions (e.g., Bergemann et al., 2017, 2018, 2019, 2020; Berger et al., 2013; Ricchi et al., 2019, 2020a, 2020b). Compositional (e.g. Th/U; Grand’Homme et al. 2016a) and age zoning in hydrothermal monazite is attributed to re-equilibration at different P-T-X conditions, initiated by tectonic events. They are thus considered as syntectonic growth domains. Fissure monazite growth starts characteristically towards the end of the quartz growth, as shown by a well-investigated Alpine fissure from Zinggenstock in the Aar Massif (Mullis, 1995, 1996), where monazite occurs as solid inclusion in late quartz and started crystallizing at ≤ 380 °C. Most common is growth of monazite on the surface of quartz or adularia. This suggests that monazite generally provides a minimum age for fissure formation, except where its crystallization can be directly linked to the formation of a new fissure generation (e.g. Bergemann et al., 2017, 2019; Ricchi et al. 2020a).

**Fig. 2:**
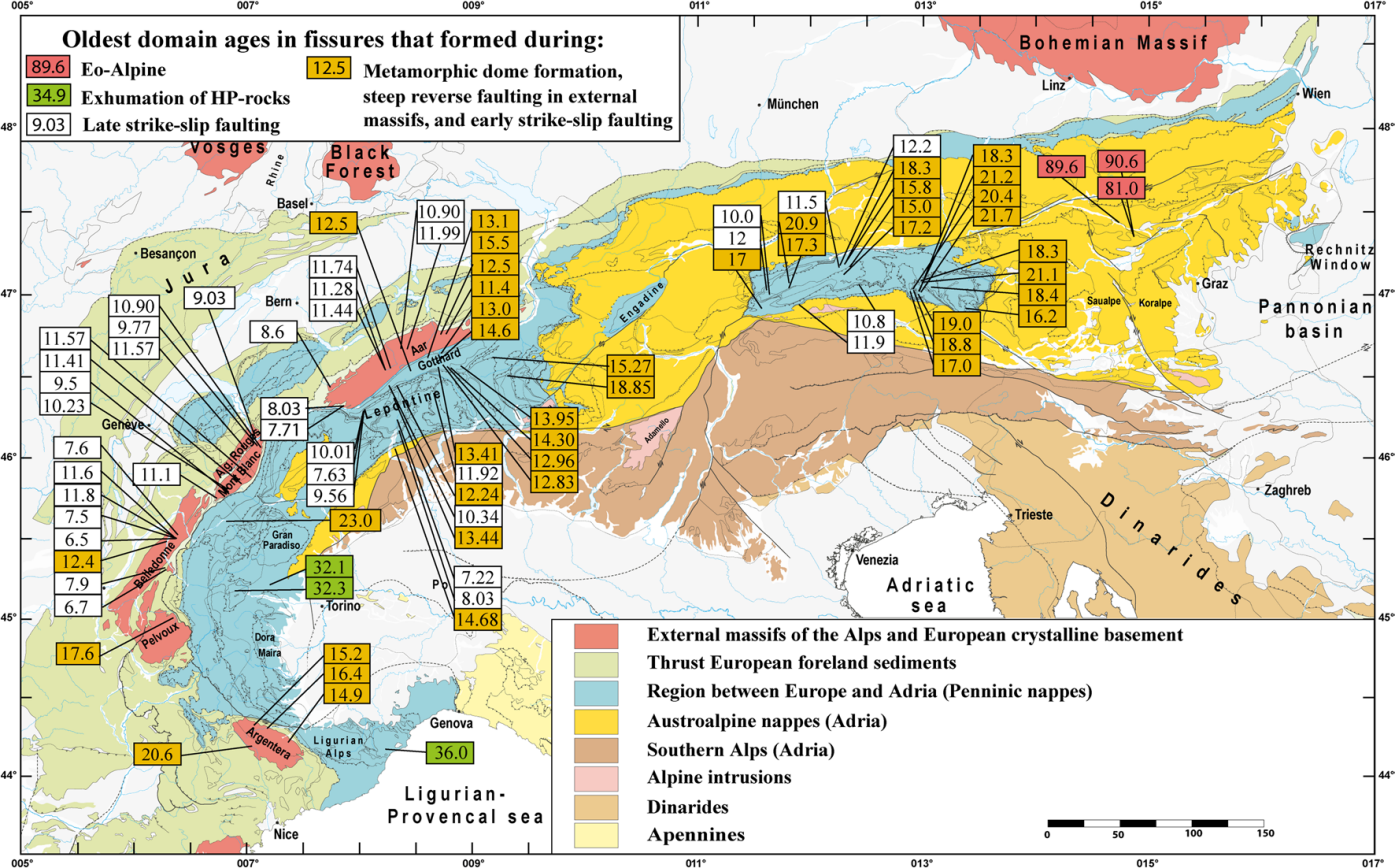
Simplified tectonic map of the Alps (based on Schmid et al., 2004; Bousquet et al., 2012b) showing the oldest growth domain age of dated fissure monazite. Note eo-Alpine (Cretaceous) ages of samples located east of the Tauern Window. Data are from Gasquet et. al. (2010), Janots et. al. (2012), Berger et. al. (2013), Gnos et. al. (2015); Grand’Homme et. al. (2016a, 2016b), Bergemann et. al. (2017, 2018, 2019), and Ricchi et. al. (2019, 2020a, 2020b)

Beneath ~ 270 °C (methane and higher hydrocarbon (HHC) fluid zones; Fig. 1; see below), tectonic movements may cause monazite or florencite (CeAl_3_(PO_4_)_2_(OH)_6_) growth. The latter has been reported from fissures in the Urals (e.g., Repina, 2010). Neither monazite nor florencite have been found in Alpine open fissures in the methane or HHC zones (Fig. 1). For this reason, crystallization ages are lacking for these fluid zones (Figs. 1 and 2).

A compilation of the oldest growth domains of dated fissure monazite is given in Fig. 2. These ages will be used here as a proxy for distinguishing episodes of fissure formation in different parts of the Alps since geochronological systems permitting a direct dating of fissure formation are lacking. Thus intersecting temperatures derived from K/Na thermometry of the earliest fluid inclusion population (Poty et al., 1974) with the regional cooling path remains a good approach to estimate the fissure formation age (e.g., Mullis, 1996).

A compilation of best estimates for σ3 axes of paleostress orientations in the Alps, based on brittle deformation data obtained by many authors was provided in Bertrand and Sue (2017). These data can be compared with measured fissure orientations.

The aim of this study is to combine published information from fissure monazite dating, quartz morphology and fissure orientation in order to attribute existing quartz fluid inclusion data to different tectono-metamorphic episodes of fissure formation.

## Tectonic and metamorphic setting of Alpine fissures

The European Alps, consisting of the E–W trending Eastern Alps and the arcuate Western Alps are the result of two orogenies. The first is Cretaceous in age, and the second Cenozoic (e.g., Froitzheim et al., 1996). The Alps are characterized by a deformed stack of large-scale nappes derived from the Adriatic continental plate, the Alpine Tethys ocean (including the Briançonnais continental basement) and the European continental plate (e.g., Schmid et al., 2004; Schuster, 2015; Stampfli et al., 1998). During both orogenies the metamorphic peak followed nappe stacking and was followed by exhumation of metamorphic rocks (e.g., Engi et al., 2004; Oberhänsli et al., 2004; Schuster et al., 2004). The Cretaceous orogeny can be studied inside the Austroalpine and parts of the Austroalpine/Alpine Tethys contacts of the Eastern Alps (e.g., Schmid et al., 2004; Fig. 1). An overview of the age range of the eo-Alpine (Cretaceous) and Alpine (Neogene) peak metamorphism is provided in Bousquet et. al. (2012a), and for the subduction related HP-LT metamorphism in Thöni (1999) and Berger and Bousquet (2008).

Abundant deformed quartz veins in metasedimentary rocks indicate that fissure formation is frequent during prograde metamorphism (e.g., Heijboer et al., 2003a; Mullis & Tarantola, 2015; Yardley, 1983), but fissures generally do not remain open and reactive in such rocks during progressive deformation and are eventually transposed into the foliation plane, and monazite enclosed in quartz cannot react with the fluid anymore. Open fissures that may reach dimensions of meter-sized clefts (e.g., Stalder et al., 1998) formed during peak to retrograde metamorphism. In the field, open fissures developing roughly perpendicular to foliation and lineation are most common at higher grade. At lower grade en-echelon fissures may occur (Mullis, 1974, 1976b).

Tectonic movements causing disequilibrium between host rock, mineralizing fluid and cleft minerals lead to dissolution/precipitation cycles (Bergemann et al., 2018, 2019, 2020; Grand’Homme et al., 2016a, 2016b; Mullis, 1976a, 2011; Mullis & De Capitani, 1997; Mullis et al., 2001a, 2001b; Ricchi et al., 2019, 2020a, 2020b; Wolf et al., 2015). In many cases, tectonic activity also leads to cracking and deformation of cleft minerals or detachment of fragments from the rock wall (Mullis, 1996, 2011; Mullis et al., 2001a, 2001b; Wolf & Mullis, 2012). Primary/secondary fluid inclusion populations are not only comparable within one cleft, but are also regionally clustered (Barlier, 1974; Frey et al., 1980a, 1980b; Mullis, 1976a, 1979, 1983, 1987; Poty, 1969; Poty et al., 1974; Stalder & Touray, 1970).

## Fissure- and cleft-filling fluid

Detailed studies by Barlier (1974), Mullis (1979, 1983), Frey et. al. (1980a, 1980b), Mullis et. al. (1994), and Rauchenstein-Martinek et. al. (2016) have shown that earliest fluid entrapped in fluid inclusions in fissure quartz, but also in metamorphic quartz (e.g., Touret, 1980), changes systematically with the regional metamorphic grade. The fluid inclusion studies also showed that crystallization in open fissures in amphibolite facies rocks or in retrogradely overprinted high-pressure rocks only formed under retrograde greenschist facies conditions.

A fluid composition zonation through the Central Alps could be established by Frey et. al. (1980a, 1980b), Mullis (1983) and Mullis et. al. (1994): at low- and medium-grade diagenetic conditions the saline aqueous fluid entrapped in fissure quartz is characterized by ~ 1 to > 80 mol% higher hydrocarbons (HHC zone; Barlier, 1974; Frey et al., 1980a, 1980b; Mullis et al., 1994; Touray et al., 1970), indicating formation temperatures and pressures of ≤ 200 °C and ≤ 0.12 GPa respectively (Mullis, 1979). In the upper diagenetic and low-grade anchizone, fluid inclusion composition is generally dominated by ~ 1 to ≥ 90 mol% methane (CH_4_ zone; Barlier, 1974; Frey et al., 1980a, 1980b; Mullis, 1976a, 1979, 1983; Mullis et al., 1973, 1994; Stalder & Touray, 1970). These methane-bearing to methane-rich fluid inclusions were trapped at temperatures and pressures between ≥ 200 and 270 ± 5 °C and between ≥ 0.12 and ≤ 0.18 GPa, respectively (Mullis, 1979, 1987; Tarantola et al., 2009). The metamorphic grade/fluid zonation correlation has been verified for very low-grade conditions by comparing fluid inclusion homogenisation temperatures with illite crystallinity and vitrinite reflection measurements in diagenetic and low-grade anchizonal terrains between 50 and 270 °C in the external parts of the Central Alps (Mullis et al., 2002, 2017). Upper anchizone, greenschist (and greenschist-facies overprinted blueschist and eclogite) facies regions are dominated by saline aqueous fluid (H_2_O-zone) (~ 80 to > 99 mol% H_2_O; Frey et al., 1980a, 1980b; Mullis et al., 1994; Poty, 1969; Poty & Stalder, 1970; Poty et al., 1974; Rauchenstein-Martinek et al., 2016). Finally, upper greenschist, amphibolite (and amphibolite-facies overprinted eclogite) facies regions are dominated by saline aqueous-carbonic fluid (CO_2_ zone) (~ 10 to > 60 mol% CO_2_; Frey et al., 1980a; Hasenberger, 1996; Heijboer et al., 2006; Kandutsch, 1989; Mullis, 1983; Mullis et al., 1994; Proce et al., 2015; Rauchenstein-Martinek, 2014; Rauchenstein-Martinek et al., 2016; Touret, 1980; Weninger, 1981).

It is interesting to note that the fluid produced around the metamorphic peak during the Tauern and Lepontine dome formations also dominates the fissure fluid composition during fissure formation under retrograde metamorphic conditions. This means, that in an amphibolite facies metamorphic terrain, fissures forming under upper greenschist facies conditions are filled with a fluid that is more or less characteristic of the amphibolite facies metamorphic grade and CO_2_-enriched (Fig. 1). Fissure fluids in amphibolite facies overprinted eclogites of the Austroalpine Saualpe-Koralpe region and the Western Alps are similarly CO_2_-enriched.

Although fissure fluids are buffered by the host rock (e.g., Hoernes & Friedrichsen, 1980) and clefts usually behave as a more or less closed system upon deformation, there may be opening at times during exhumation, leading to loss or influx of fluid from external sources (e.g., Mullis, 1983, 1996; Mullis et al., 1994; Poty, 1969).

## Linking fluid type with quartz habit

**Fig. 3:**
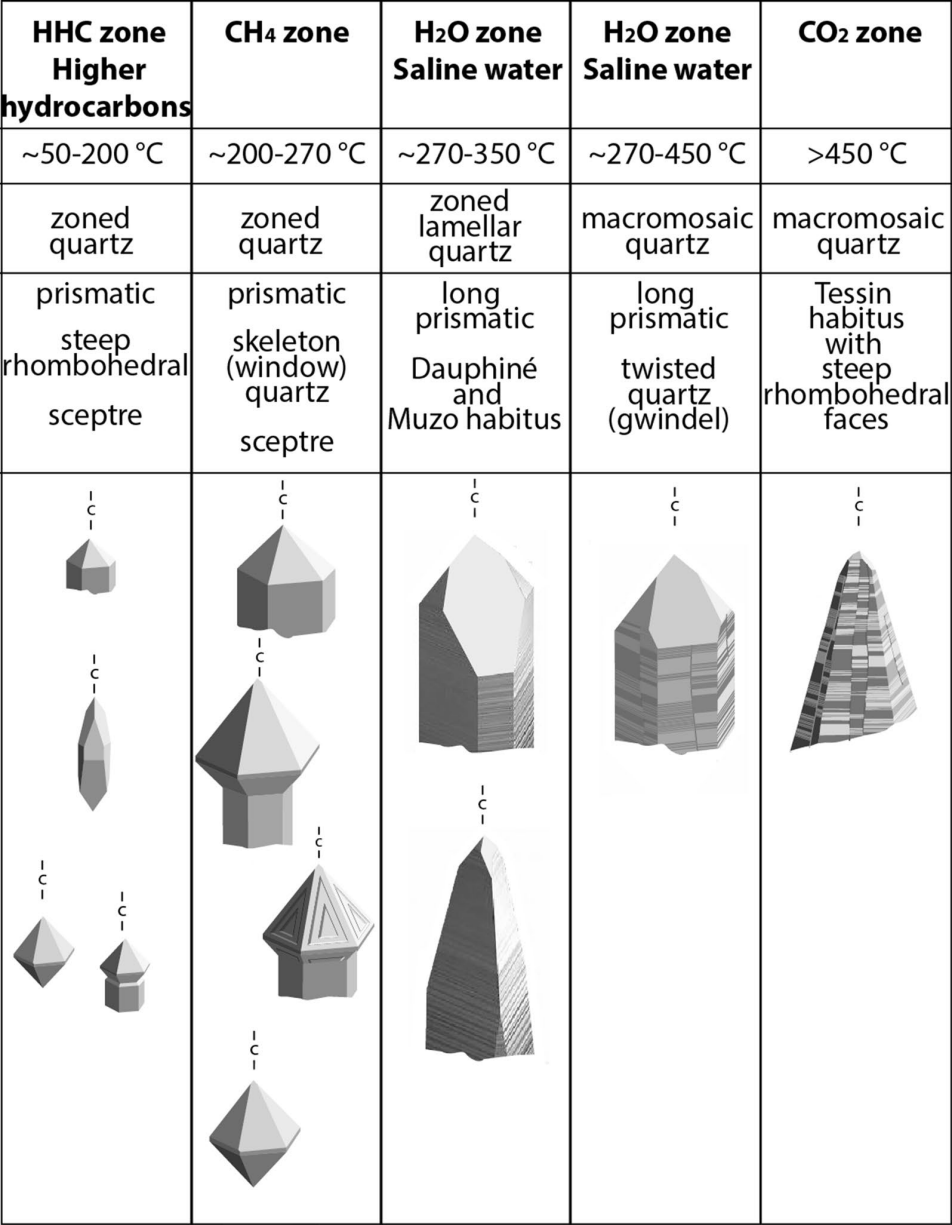
Compilation of most common fissure quartz habits as a function of fluid type (Kandutsch, 1989; Mullis & Ramseyer, 1996; Mullis et al., 1994; Mullis, 1976a, 1983, 1991; Poty, 1969; Stalder & Touray, 1970). Quartz crystals of the higher hydrocarbone zone are generally mm-sized. Note that in amphibolite facies rocks (or in eclogites overprinted by amphibolite facies) fissures and clefts form only during retrograde greenschist facies deformation. The quartz habit is a result of fluid generated at final prograde, peak- to beginning retrograde metamorphic conditions, or during the Barrow-type metamorphic overprinting of high-pressure rocks. In general, the quartz habit developing during the early growth stages of fissure quartz predominates, but retrograde quartz overgrowth is locally common (e.g., Mullis, 1976a, 1983, 1991; Mullis et al., 1994; Niedermayr, 1993; Poty, 1969; Rykart, 1995). –c– indicates the orientation of the c-axis. The quartz morphologies and temperature estimates are compiled in Poty et. al. (2007)

The prevailing fluid has also a remarkable effect on the fissure quartz habit (e.g., Kandutsch et al., 1998; Mullis et al., 1994; Mullis, 1976a, 1983, 1991; Poty, 1969; Stalder & Touray, 1970). Judging by the fluid zonation in mono-metamorphic sedimentary rocks, fissure quartz changes systematically its morphology with changing fluid zone (e.g., Mullis et al., 1994; Fig. 3) and increasing metamorphic grade of the host rock.

In the HHC zone (≤ 200 °C), freely grown quartz is short prismatic and is often overgrown by sceptre quartz. Bipyramidal quartz can also be observed. In oil rich fluids, fissure quartz tends to develop a steep rhombohedral habit (Mullis, 1991; Mullis et al., 1994; Fig. 3). All quartz crystals of the HHC zone are generally a few millimetres in size.

In the CH_4_ zone, that covers the upper diagenetic and low-grade anchizone (T ≥ 200 °C to ≤ 270 °C; Fig. 3), prismatic, sceptre (more rarely bipyramidal) and skeletal (‘window’ shaped) quartz is characteristic. Water-methane unmixing (immiscibility) causes fast growth along corners and edges, responsible for sceptre and ‘window’ shaped quartz growth (Fig. 3; Mullis, 1976a, 1991; Mullis et al., 1994).

In the H_2_O zone covering anchizonal and lower greenschist facies metamorphic conditions (≥ 270 °C to ~ 450 °C), fluids are hydrous, saline and contain < 10 mol% of dissolved CO_2_. Quartz crystallizing in such a fluid is prismatic in shape, shows complex macro-mosaic twinning (Friedlaender, 1951) and characteristic sutures on prism faces (Fig. 3).

In zones with prolonged tectonic activity, such as nappe contacts or the interface of crystalline basement rocks with the overlying metasedimentary cover of sub-greenschist metamorphic conditions (≥ 270 °C to ~ 350 °C), lamellar quartz of Dauphiné habit (Bambauer et al., 1961) characteristically forms. The Dauphiné habit is characterized by the development of one pyramid face growing much larger than the other five faces (e.g., Jourdan et al., 2009; Kandutsch & Wachtler, 2000; Kürsteiner et al., 2015; Mullis, 1980, 1991; Mullis & Ramseyer, 1996; Poty, 1969; Soom, 1986; Fig. 3). In some cases, the disappearance of prism faces towards the pyramid of quartz leads to the Muzo habit (Gansser, 1963; Fig. 3) that has been reported from several localities of the Eastern Alps (e.g. Glas, 1992; Hasenberger, 1996; Kandutsch, 1989; Kandutsch & Wachtler, 2000; Rykart, 1995).

In the CO_2_ zone Tessin habit quartz is omnipresent and characterized by the predominance of steep pyramidal faces over prism faces giving the quartz a pointed shape (Fig. 3). Characteristic sutures occur on prism/steep pyramidal faces. This habit is most pronounced in fissures of meta-sedimentary rocks that underwent high-grade greenschist or amphibolite facies metamorphism (Frey et al., 1980a; Hasenberger, 1996; Kandutsch, 1989; Kandutsch et al., 1998; Lukscheiter & Morteani 1980; Mullis, 1991, 1996; Mullis et al., 1994). Quartz in granitoid lithologies of comparable metamorphic grade and with a CO_2_ content of > 2 to < 10 mol% CO_2_ (Mullis et al., 2001a, 2001b), is generally less pointed but displays a mix between prismatic and steep pyramidal faces leading to a characteristic horizontal striation on the prism faces. Both, Tessin habit quartz and prismatic quartz with steep pyramidal faces show macro-mosaic twinning (Friedlaender, 1951) and sutures on their prism faces (Fig. 3).

Fluid immiscibility in the CO_2_-zone (Mullis, 1983; Mullis et al. 1994) and late infiltration of a CO_2_-enriched fluid in the CO_2_ and water zone (Mullis et al., 1994; Poty, 1969) at temperatures between ~ 330 and 250 °C lead to scepter quartz overgrowths (often amethyst) on both, Tessin habit and prismatic quartz, respectively (e.g., Burgsteiner, 2004; Hossfeld, 1977; Mullis, 1983; Mullis & Ramseyer, 1996; Mullis et al., 1994; Poty, 1969; Stalder et al., 1998; Wagner et al., 1972). Amethyst shows lamellar growth.

Along a section showing increasing Barrow-type metamorphic grade, this supports the preliminary conclusion that metamorphic grade, fluid composition and quartz habit (Fig. 3) are directly linked.

## Fissure monazite ages and fissure orientation

**Fig. 4:**
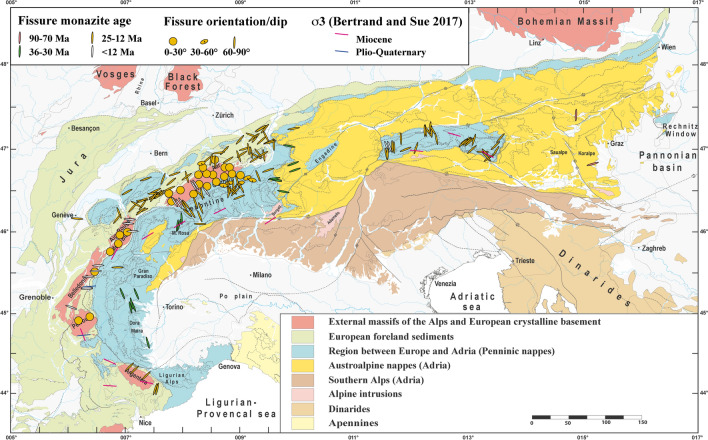
Simplified tectonic map of the Alps (based on Bousquet et al., 2012b; Schmid et al., 2004), showing characteristic fissure orientations (perpendicular to σ3) and best Miocene (red lines) and Plio-Quaternary (blue lines) σ3 axes of paleostress data obtained by Bertrand and Sue (2017). The color of the fissures is according to the oldest fissure monazite crystallization age recorded for a fissure generation. For regions lacking fissure monazite, the attribution is based on the age of the metamorphic overprinting. Some data are from Kandutsch et. al. (1998) and Sharp et. al. (2005). For discussion is referred to the text

A large and homogeneous set of cleft monazite growth domain ages covering most parts of the Alps is now available (Bergemann et al., 2017, 2018, 2019, 2020; Berger et al., 2013; Gasquet et al., 2010; Gnos et al., 2015; Grand’Homme et al. 2016a, 2016b; Janots et al., 2012; Ricchi et al., 2019, 2020a, 2020b; Fig. 2). Recalculated values for some monazites from the Aar Massif and the Gotthard Nappe are provided in the supplement (Additional file 1: Table S1 and Fig. S1). In most cases, fissure monazite crystallization does not date fissure formation, because it starts to crystallize typically < 300–350 °C (e.g., Bergemann et al., 2019; Gnos et al., 2015; Mullis, 1996; Ricchi et al.,2020a), although growth at higher temperature was recently reported (Janots et al., 2019). Monazite characteristically crystallizes a few million years after cleft formation, towards the end of the fissure quartz growth (e.g., Gnos et al., 2015; Mullis, 1996). Only in some cases was it possible to revisit the exact monazite find location to collect complementary structural data. A compilation of fissure and cleft orientations for areas where fissure monazite was reported is provided in Fig. 4. Gasquet et. al. (2010) were the first to date fissure monazite derived from an older generation of horizontal and a younger generation of vertical fissures in the Belledonne and Pelvoux massifs, using the LA-ICP-MS technique. This yielded ages between 17.6 ± 0.3 and 5.4 ± 0.5 Ma. They could show that ages between 11 and 5 Ma were related to two stages of dextral strike-slip faulting. Janots et. al. (2012) used the SIMS technique, providing higher spatial resolution, and dated growth domains in two grains from horizontal fissures located in the Aar Massif and the Gotthard Nappe. They obtained ages of 15.2 ± 0.3 to 13.5 ± 0.4 Ma. One grain showed a stage of dissolution between two growth domains. Berger et. al. (2013) and Bergemann et. al. (2017) used fissure monazite for dating a younger generation of vertical fissures in the Aar Massif at 11.75 ± 0.41 to 6.32 ± 0.20 Ma and could show that dextral strike-slip faulting in the Aar Massif occurred roughly coeval with strike-slip faulting in the Belledonne Massif (Fig. 1). Systematic studies by Bergemann et. al. (2019, 2020) covering the Mont Blanc/Aiguilles Rouges massifs and the Lepontine Dome and by Ricchi et. al. (2019, 2020a) covering the Aar Massif/Gotthard Nappe and the entire Tauern Window confirmed the widespread formation of late, vertical fissures that formed in association with sinistral (Tauern Window) and dextral strike-slip faulting (Simplon-Rhone fault and faults in the Aar, Gotthard, Mont Blanc, Aiguilles Rouges, Belledonne and Pelvoux massifs) starting at ~ 12 Ma. Sinistral strike-slip faulting in the Tauern Window resulted in vertical fissures (Ricchi et al., 2020a), oriented subparallel to the older generation (Fig. 4). Coeval dextral strike-slip faults in the Western Alps have corresponding vertical fissures that strike SE in the Aar Massif and E–W in the Mont Blanc, Aiguilles Rouges, Belledonne and Pelvoux massifs (Fig. 4). From this time on, episodic reactivation of the strike-slip faults occurred (Bergemann et al., 2017, 2019; Berger et al., 2013; Gasquet et al., 2010; Ricchi et al., 2019, 2020a, 2020b; Schneider et al., 2013), with the youngest recorded fissure monazite domain age is ~ 5 Ma (Bergemann et al., 2020). Present day faulting shows the same kinematics and inferred stress field, indicating constant stress fields over the last several millions of years (e.g., Bertrand & Sue, 2017).

In the Tauern and Lepontine metamorphic domes, fissure formation is very common during extensional unroofing, and the oldest growth domains record an age range of 21.7 ± 0.4 to 11.92 ± 0.26 Ma (Bergemann et al., 2020; Gnos et al., 2015; Ricchi et al., 2020a; Fig. 2). Fissures are vertical, N to NE striking in the Eastern, and SE striking in the Western Tauern Window and mostly NW striking in the Lepontine structural and metamorphic dome (Fig. 4).

At the same time fissure monazite crystallization also occurred in the external massifs and, except for Argentera, in association with reverse faulting (Bergemann et al., 2019; Gasquet et al., 2010; Janots et al., 2012; Ricchi et al., 2019). For this reason, fissures of this generation are oriented roughly horizontal in the Aar Massif/Gotthard Nappe, the Mont Blanc/Aiguilles Rouges massifs and the Belledonne/Pelvoux massifs (Fig. 4).

The orientation of early vertical fissures in high-pressure regions of the Western Alps differs from those of the external massifs and the Lepontine dome. The fissures were dated to 36.6 ± 0.6 Ma to 30.2 ± 0.5 Ma in the Briançonnais Unit and strike regionally quite homogenously SE (Grand’Homme et al. 2016a; Ricchi et al., 2020b; Fig. 4). However, the fissures located just southwest of the Simplon detachment fault may have been affected by younger tectonic movements during the Lepontine dome formation.

Vertical fissures associated with dextral strike-slip faults are E striking in the Briançonnais and ENE striking in the Argentera Massif (Fig. 4). These fissures are older than those forming after ~ 12 Ma during dextral strike-slip faulting in all other external massifs.

At the other end of the Alps, much older, eo-Alpine fissure monazites were found in Austroalpine units located east of the Tauern Window, where monazite crystallization occurred around 90 Ma in association with strike-slip faulting (Bergemann et al., 2018), during exhumation of the Koralpe-Saualpe region. These fissure monazites partially recrystallized in association with extensional tectonics associated with the formation of the Gosau basins between ~ 90 and 70 Ma.

In general, very few data exist for the Austroalpine units and no data are available for the region west of the Tauern Window. In the Koralpe-Saualpe region Cretaceous fissures are vertical and roughly ENE striking, whereas north of it, in the Greywacke Zone, they also show a N–S orientation (Fig. 4).

Monazite ages have been compiled in Fig. 2 (see figure caption for references). They are attributed to fissure generations that formed: (1) During exhumation of different Austroalpine units in the Cretaceous (eo-Alpine), (2) during exhumation of the Penninic high pressure units, (3) during metamorphic dome formation, steep reverse faulting in the external massifs and during early strike-slip faulting affecting the high pressure units of the Western Alps and the Argentera Massif, and (4) during late strike-slip faulting affecting the metamorphic dome regions and all external massifs, except Argentera. All available monazite crystallization domain ages and spot age ranges are compiled in Table 1. A compilation of fissure and cleft orientations for areas where fissure monazite was reported is provided in Fig. 4.

**Table 1:** Fissure monazite ^208^Pb/^232^Th domain and spot age ranges

Sample	Domain age	Domain age	Domain age	Domain age	Domain age	Spot age range	References
Eastern Alps							
T5	90.6 ± 3.6	89.2 ± 1.8	78.3 ± 1.9			92.78 ± 2.49 to 71.05 ± 1.96	Bergemann et. al. (2018)
RAHM2	80.7 ± 1.6	81.0 ± 1.5				84.20 ± 2.21 to 71.19 ± 2.09	Bergemann et. al. (2018)
SCHO1	89.6 ± 1.9	75.5 ± 1.7	73.4 ± 2.6			95.15 ± 2.52 to 75.16 ± 2.14	Bergemann et. al. (2018)
Tauern window							
TAUERN1	18.99 ± 0.55	17.56 ± 0.55	16.25 ± 0.55	15.00 ± 0.51		18.48 ± 0.54 to 14.53 ± 0.52	Gnos et. al. (2015)
TAUERN2	15.12 ± 0.48					15.44 ± 0.43 to 14.96 0.63	Gnos et. al. (2015)
TAUERN3	18.06 ± 0.42	17.18 ± 0.49	15.49 ± 0.15			18.53 ± 0.44 to 15.29 ± 0.37	Gnos et. al. (2015)
TAUERN4	15.56 ± 0.70					15.98 ± 0.39 to 14.81 ± 0.36	Gnos et. al. (2015)
INNB1						11.52 ± 0.2 to 10.4 ± 0.2	Ricchi et. al. (2020a)
ZEI1	10.0 ± 0.2					10.8 ± 0.3 to 7.2 ± 0.2	Ricchi et. al. (2020a)
SCHR1	20.9 ± 0.6	20.3 ± 0.2	19.7 ± 0.4			21.9 ± 0.5 to 19.3 ± 0.5	Ricchi et. al. (2020a)
MAYR4						11.8 ± 0.2 to 8.9 ± 0.2	Ricchi et. al. (2020a)
PFIT1	17.3 ± 0.3	13.2 ± 0.3				17.8 ± 0.4 to 12.9 ± 0.3	Ricchi et. al. (2020a)
BURG2						17.1 ± 0.4 to 12.1 ± 0.3	Ricchi et. al. (2020a)
PLAN1	11.9 ± 0.2					12.6 ± 0.3 to 7.8 ± 0.2	Ricchi et. al. (2020a)
SCHEI1	18.3 ± 1.1	17.4 ± 0.4	16.6 ± 0.2			18.9 ± 0.5 to 15.9 ± 0.4	Ricchi et. al. (2020a)
HOPF2	12.2 ± 0.4	12.2 ± 0.5				13.7 ± 0.4 to 11.0 ± 0.3	Ricchi et. al. (2020a)
GART1	16.3 ± 0.2					16.9 ± 0.3 to 14.5 ± 0.4	Ricchi et. al. (2020a)
NOWA3	15.8 ± 0.5	14.9 ± 1.1				17.0 ± 0.2 to 13.8 ± 0.8	Ricchi et. al. (2020a)
GART3	15.5 ± 0.5					16.1 ± 0.4 to 12.0 ± 0.4	Ricchi et. al. (2020a)
STEI2	17.2 ± 0.2					17.5 ± 0.4 to 16.8 ± 0.4	Ricchi et. al. (2020a)
KNOR1	10.8 ± 0.3	10.6 ± 0.3	10.4 ± 0.2			11.6 ± 0.4 to 10.8 ± 0.3	Ricchi et. al. (2020a)
KAIS6	21.2 ± 0.5	20.9 ± 0.2	20.6 ± 0.5	18.8 ± 0.5		22.1 ± 0.6 to 17.6 ± 0.6	Ricchi et. al. (2020a)
SALZ18	18.3 ± 0.4					19.5 ± 0.5 to 15.8 ± 0.4	Ricchi et. al. (2020a)
LOHN4	21.1 ± 0.2	18.4 ± 0.6				22.9 ± 0.6 to 17.3 ± 0.6	Ricchi et. al. (2020a)
ORT1	18.4 ± 0.3					19.0 ± 0.6 to 17.0 ± 0.7	Ricchi et. al. (2020a)
EUKL2	21.7 ± 0.4					22.3 ± 0.5 to 21.2 ± 0.5	Ricchi et. al. (2020a)
HOAR1	20.4 ± 0.2	19.9 ± 0.3				21.2 ± 0.7 to 19.0 ± 0.9	Ricchi et. al. (2020a)
MOKR1	18.8 ± 0.5					22.6 ± 0.4 to 14.4 ± 0.2	Ricchi et. al. (2020a)
SAND1	17.0 ± 0.8					22.0 ± 0.3 to 14.7 ± 1.0	Ricchi et. al. (2020a)
REIS1	16.2 ± 0.5	13.6 ± 0.6				17.8 ± 0.6 to 13.5 ± 0.8	Ricchi et. al. (2020a)
Lepontine							
VANI 6	14.68 ± 0.47					16.80 ± 0.31 to 10.62 ± 0.18	Bergemann et. al. (2020)
BETT 11	9.96 ± 0.30	7.53 ± 0.31				10.55 ± 0.33 to 7.34 ± 0.26	Bergemann et. al. (2020)
DURO 1						10.82 ± 0.26 to 8.21 ± 0.20	Bergemann et. al. (2020)
DURO 2	7.63 ± 0.13	7.18 ± 0.18				11.48 ± 0.28 to 7.02 ± 0.18	Bergemann et. al. (2020)
DUTH 6	11.92 ± 0.26	9.74 ± 0.22				12.60 ± 0.37 to 9.33 ± 0.32	Bergemann et. al. (2020)
GRAESER 1	11.88 ± 0.23	10.18 ± 0.24	8.93 ± 0.14	7.73 ± 0.17		12.14 ± 0.30 to 7.57 ± 0.19	Bergemann et. al. (2020)
GRAESER 3						15.60 ± 0.61 to 6.36 ± 0.39	Bergemann et. al. (2020)
GRAESER 4						12.25 ± 0.51 to 11.88 ± 0.47	Bergemann et. al. (2020)
KLEM 1	10.43 ± 0.24	9.47 ± 0.18	8.36 ± 0.17			10.64 ± 0.26 to 7.97 ± 0.20	Bergemann et. al. (2020)
KLEM 2	13.44 ± 0.32	11.81 ± 0.30	10.16 ± 0.28			13.65 ± 0.33 to 9.47 ± 0.40	Bergemann et. al. (2020)
KLEM 3	12.24 ± 0.35	8.9 ± 1.2				12.96 ± 0.46 to 8.43 ± 0.32	Bergemann et. al. (2020)
SCHIESS 1	9.56 ± 0.25	7.02 ± 0.23				9.94 ± 0.25 to 6.78 ± 0.18	Bergemann et. al. (2020)
VANI 4	8.03 ± 0.44					9.27 ± 0.43 to 6.89 ± 0.37	Bergemann et. al. (2020)
VANI 5	7.22 ± 0.27	5.27 ± 0.31				8.07 ± 0.36 to 4.86 ± 0.24	Bergemann et. al. (2020)
BLAS 1	12.83 ± 0.39					14.49 ± 0.26 to 7.82 ± 0.22	Bergemann et. al. (2020)
DUTH 2	13.41 ± 0.70					14.34 ± 0.41 to 11.15 ± 0.43	Bergemann et. al. (2020)
DUTH 3	13.95 ± 0.33	12.73 ± 0.35	10.95 ± 0.33			14.53 ± 0.43 to 10.61 ± 0.34	Bergemann et. al. (2020)
LUCO 1	14.30 ± 0.21	10.14 ± 0.42				14.74 ± 0.30 to 9.90 ± 0.17	Bergemann et. al. (2020)
SALZ 2	12.92 ± 0.25	10.87 ± 0.27				14.28 ± 0.74 to 10.51 ± 0.39	Bergemann et. al. (2020)
TAMB 1	18.85 ± 0.77	17.37 ± 0.42	14.95 ± 0.70	13.08 ± 0.32		19.02 ± 0.47 to 12.32 ± 0.52	Bergemann et. al. (2020)
VALS	15.27 ± 0.35	14.77 ± 0.42	14.70 ± 0.41	13.80 ± 0.49	12.94 ± 0.49	16.43 ± 0.61 to 12.09 ± 0.57	Bergemann et. al. (2020)
Aar Massif and Gotthard Nappe							
GRIESS1	14.7 ± 0.5	13.8 ± 0.2				16.0 ± 1.0 to 12.9 ± 0.5	Janots et. al. (2012)
BLAU1	15.5 ± 0.2	15.2 ± 0.3	13.6 ± 0.4			15.9 ± 0.8 to 13.2 ± 0.4	Janots et. al. (2012)
BALT2	8.03 ± 0.22	6.60 ± 0.18	6.32 ± 0.20			8.05 ± 0.22 to 6.17 ± 0.16	Berger et. al. (2013)
BALT4	7.71 ± 0.40	7.40 ± 0.17	6.49 ± 0.25	6.25 ± 0.60		9.18 ± 0.30 to 6.11 ± 0.15	Berger et. al. (2013)
PK2	11.61 ± 0.29	11.35 ± 0.34	10.85 ± 0.36			11.75 ± 0.41 to 10.55 0.42	Bergemann et. al. (2017)
GRIM4	11.44 ± 0.23	11.15 ± 0.19				11.69 ± 0.29 to 11.07 ± 0.27	Bergemann et. al. (2017)
GRIM3	11.21 ± 0.22	11.28 ± 0.22	7.02 ± 0.31			11.66 ± 0.29 to 6.75 ± 0.17	Bergemann et. al. (2017)
NEAT1^#^	11.4 ± 0.2					11.5 ± 0.3 to 6.8 ± 0.2	Ricchi et. al. (2019)
JOLI2^#^						8.2 ± 0.2 to 5.6 ± 0.1	Ricchi et. al. (2019)
GAST1^#^	8.6 ± 0.1					9.9 ± 0.2 to 7.2 ± 0.2	Ricchi et. al. (2019)
GUTT1						14.24 ± 0.94 to 8.68 ± 0.62	Ricchi et. al. (2019)
GOSCH1	11.99 ± 0.56					12.92 ± 0.45 to 8.94 ± 0.35	Ricchi et. al. (2019)
SALZ21	10.90 ± 0.29	10.63 ± 0.29				11.38 ± 0.34 to 8.36 ± 0.29	Ricchi et. al. (2019)
MUTT1^#^	12.5 ± 0.6	12.0 ± 0.5				13.2 ± 0.3 to 10.2 ± 0.3	Ricchi et. al. (2019)
UNTE1^#^	14.6 ± 0.4					15.6 ± 0.4 to 11.0 ± 0.4	Ricchi et. al. (2019)
CAVR1^#^	13.0 ± 0.3					14.2 ± 0.2 to 10.4 ± 0.2	Ricchi et. al. (2019)
GOTT1^#^	13.1 ± 0.2					13.8 ± 0.3 to 11.6 ± 0.3	Ricchi et. al. (2019)
Mont Blanc and Aiguilles Rouges							
VDG*	11.1 ± 0.2					11.4 ± 0.6 to 10.7 ± 0.7	Grand’Homme et. al. (2016a)
AIGG1	10.23 ± 0.41					13.94 ± 0.83 to 9.47 ± 0.44	Bergemann et. al. (2019)
BLANC2	9.50 ± 1.10	8.12 ± 0.79				10.94 ± 0.57 to 7.16 ± 0.34	Bergemann et. al. (2019)
CAT2	9.77 ± 0.23	8.12 ± 0.79				12.90 ± 1.09 to 7.20 ± 0.33	Bergemann et. al. (2019)
HEL1	11.41 ± 0.50	11.14 ± 0.20	10.92 ± 0.39	8.24 ± 0.38		11.95 ± 0.30 to 7.69 ± 0.22	Bergemann et. al. (2019)
SALZ10	9.03 ± 0.19	7.91 ± 0.26				9.34 ± 0.33 to 7.23 ± 0.37	Bergemann et. al. (2019)
SALZ11	11.57 ± 0.30	11.42 ± 0.23				11.96 ± 0.47 to 7.76 ± 0.34	Bergemann et. al. (2019)
SALZ14	10.90 ± 0.50	9.64 ± 0.23	6.71 ± 0.23			11.29 ± 0.35 to 6.58 ± 0.15	Bergemann et. al. (2019)
SALZ15	9.17 ± 0.26					9.90 ± 1.38 to 6.58 ± 0.33	Bergemann et. al. (2019)
Belledonne and Pelvoux Massifs							
EDR1*	11.7 ± 0.2						Gasquet et. al. (2010)
EDR3*	11.5 ± 0.7						Gasquet et. al. (2010)
EDR4*	11.3 ± 0.7						Gasquet et. al. (2010)
MoE*	6.7 ± 0.9						Gasquet et. al. (2010)
MoB*	7.2 ± 0.4						Gasquet et. al. (2010)
MoG*	5.4 ± 0.5						Gasquet et. al. (2010)
Pdl*	17.6 ± 0.3						Gasquet et. al. (2010)
RDN*	10.3 ± 0.5	8.3 ± 0.1				11.7 ± 0.8 to 8.1 ± 0.4	Grand’Homme et. al. (2016a)
LCO*	7.6 ± 0.2					8.6 ± 1.4 to 7.2 ± 0.8	Grand’Homme et. al. (2016a)
KER*	7.5 ± 0.3					8.1 ± 0.4 to 7.1 ± 0.5	Grand’Homme et. al. (2016a)
SOY*	11.8 ± 0.1	7.5 ± 0.2				12.2 ± 0.5 to 7.6 ± 0.5	Grand’Homme et. al. (2016a)
E2R*	12.4 ± 0.1					13.0 ± 0.5 to 12.1 ± 0.5	Grand’Homme et. al. (2016a)
SAV*	6.7 ± 0.1					7.1 ± 0.4 to 6.5 ± 0.3	Grand’Homme et. al. (2016a)
SMC*	7.9 ± 0.3	6.3 ± 0.2				8.4 ± 0.5 to 6.0 ± 0.5	Grand’Homme et. al. (2016a)
Argentera Massif							
ISO*	20.6 ± 0.3					22.0 ± 0.9 to 20.2 ± 0.8	Grand’Homme et. al. (2016a)
MORI1	15.2 ± 0.3	15.1 ± 0.4				15.9 ± 0.4 to 14.2 ± 0.3	Ricchi et. al. (2020b)
VINA1	16.4 ± 0.2	15.3 ± 0.3	14.4 ± 0.5			16.7 ± 0.3 to 13.0 ± 0.2	Ricchi et. al. (2020b)
GESS1	14.9 ± 0.3					16.2 ± 0.4 to 12.1 ± 0.3	Ricchi et. al. (2020b)
Briançonnais and Piemontais							
MTC*	32.3 ± 0.3					32.9 ± 1.4 to 31.6 ± 1.2	Grand’Homme et. al. (2016a)
MTV*	23.0 ± 0.3					23.9 ± 0.9 to 22.0 ± 0.8	Grand’Homme et. al. (2016a)
VIU1	30.2 ± 0.5					33.2 ± 0.5 to 28.5 ± 0.5	Ricchi et. al. ( 2020b)
BALZI2	36.0 ± 0.6	30.3 ± 0.5	25.4 ± 0.5			50.1 ± 0.6 to 11.2 ± 0.6	Ricchi et. al. ( 2020b)

Ages marked with * were obtained by LA-ICP-MS, all other data by ion microprobe. Ages marked with ^#^ differ from Ricchi et. al. (2019) due difference in data reduction (see Additional file 1 for detailed explanation)

## Discussion

The formation of open fissures and fissure monazite crystallization occurs in the upper crust at conditions at or below 450–550 °C and 0.3–0.6 GPa (e.g., Diamond & Tarantola, 2015; Heijboer et al., 2003a, 2006;Mullis, 1974, 1976a, 1976b, 1983, 1991, 1996, 2011; Mullis & Tarantola, 2015; Mullis et al., 1994; Poty, 1969; Poty et al., 2007, 2018; Rauchenstein-Martinek et al., 2016; Sharp et al., 2005), in association with regional scale deformation or localized faulting.

### Fluid inclusion type, metamorphic grade and quartz habit

In most open fissures the fluid derived from the surrounding rock and formed at or near the peak of metamorphism. For this reason it changes systematically with regional metamorphic grade (Fig. 1), causing a change in the quartz habit with changing fluid zones. There are cases (e.g., Bedretto Valley, Ticino; Mullis, 1991) where late tectonic movements produced fissures that are oriented parallel to an earlier generation, but can easily be recognized due to its different quartz habit. Such occurrences help to recognize discrete faults, where the corresponding lineation is only developed on few, narrow fault planes. Complemented with fluid inclusion thermometry (Fig. 3) the quartz habit is very useful for constraining the temperature of formation of the fissure.

### Alpine fissure formation over time

The Alps are the result of two major orogenies (e.g., Froitzheim et al., 1996), Cretaceous and Cenozoic in age. Different Alpine units underwent exhumation at different times, and hence, fissure formation occurred in association with different tectonic settings. Crosscutting fissure relationships may locally be found in the external massifs (Aar, Gotthard, Mont Blanc, Aiguilles Rouges, Belledonne, Pelvoux (e.g., Ricchi et al., 2019; Steck, 1968). In rare cases, multiple deformation results in complex fissure shapes or intersecting fissures (e.g., Ricchi et al., 2019; Steck, 1968). In other areas, fissures that formed at different episodes show subparallel orientations. Meaning that often only the study of the surrounding rock fabric, in combination with the deformation history, may provide additional clues about the formation and evolution of a fissure. Due to such tectonic reactivations, fissure mineral growth is thus in many cases the result of multiple, more or less pronounced, regional deformation events (Burkhard & Kerrich, 1988; Heijboer et al., 2003b; Mullis, 1975, 1976b, 1996, 2011; Mullis et al., 2001a, 2001b; Wolf & Mullis, 2012).

P–T estimates based on quartz fluid inclusions indicate that in the higher hydrocarbon and methane fluid inclusions zones (Fig. 1) and in parts of the water zone located in lower greenschist facies rocks, fissure formation occurred roughly coeval with the metamorphic peak. This concerns the thrust European foreland, Helvetic nappes, the most external parts of the External Massifs, and probably also parts of the Australpine regions (Fig. 1). Fissures located in higher grade greenschist and amphibolite facies rocks in the water and CO_2_ fluid zones formed after nappe stacking and metamorphic peak under retrograde greenschist facies conditions. This concerns large parts of the External Massifs, the Penninic nappes, and in the Austroalpine nappes the Koralpe-Saualpe region and probably also other regions. In strike-slip settings, fissure formation is related to tectonic activity under low grade greenschist facies to very-low grade metamorphic conditions.

The quartz fluid inclusion map shown in Fig. 1 shows that fissures are rare in some regions, but abundant in others. In the Austroalpine units, “rareness” may be linked to the lack of knowledge of discovered and investigated fissures, weathering and vegetation cover. The rareness of clefts in the southeastern sector of the Lepontine dome (Fig. 1) indicates that fluids were either efficiently drained or not available on the melt bearing level of the dome (e.g., Burri et al., 2005), or the area highly overprinted due to large and non-localized deformation in this region. The latter is most likely, due to the frequent high-grade quartz veins in this area (i.e., sillimanite/kyanite-quartz veins; Allaz et al., 2005; Beitter et al., 2008), possibly indicating fluid escape in veins at amphibolite conditions.

**Fig. 5:**
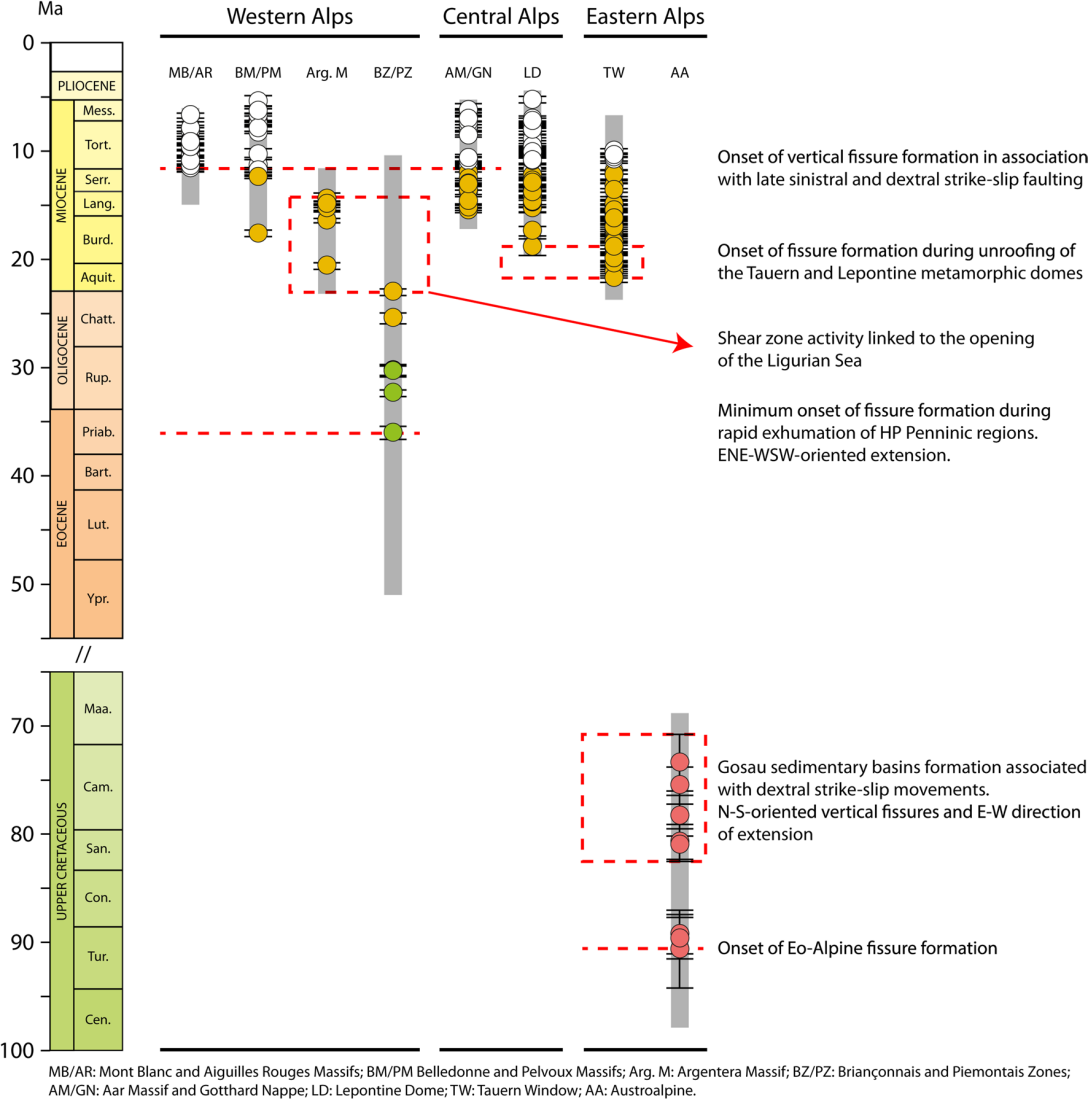
Compilation of fissure monazite domain ages (dots with error bars) for different Alpine units. The vertical grey bars indicate the spot age range. Important events leading to fissure formation are marked with dashed red lines and boxes

Fissure monazite dating shows that fissure formation in association with metamorphism and tectonic movements occurred several times during the Alpine evolution (Fig. 5). In comparison with published orogenic timetables of the Alps (e.g., Pfiffner (2015) and references therein, fissure formation occurred after main thickening and nappe development, mainly during post-nappe deformation. These post-nappe deformation stages were mainly related to exhumation processes. A special example of exhumation process occurred in the Tauern Window, where large scale folding was contemporaneous with normal faulting and strike-slip movements (Rosenberg et al., 2018). The vertical fissures developed mainly during these movements (Brenner-Katschberg phase in Pfiffner (2015)). Post-nappe deformation in the Central Alps included large scale folding and normal faulting (e.g., Chièra folding, Forcola- and Simplon normal faulting). In the external massifs (Aar Massif/Gotthard Nappe, Mont Blanc/Aiguilles Rouges Massifs and Belledonne/Pelvoux Massifs) fissure formation occurred during phases of dominant reverse faulting (e.g., Handegg deformation phase in the Aar Massif; Herwegh et al., 2020; Ricchi et al., 2019). This phase is related to the well-known post-nappe deformation of the Helvetic units (Ruchi phase in the east, Kiental phase in the west; Pfiffner 2011, 2015). In the very-low grade metamorphic European foreland sediments fissures show two main orientations (Fig. 4). Their formation is related to late detachments and extensional movements in association with the exhumation of the external massifs. In the high-pressure dominated regions of the Western Alps, fissure formation occurred also during exhumation and Barrow-type metamorphic overprinting of the high-pressure units. In the Austroalpine units, eo-Alpine fissures formed in the Koralpe-Saualpe region during the exhumation following crustal thickening and Barrow-type metamorphism. The orientation (Fig. 4) indicates that they formed during the D3 deformation phase of Kurz and Fritz (2003). Fissures that formed in association with strike-slip faulting belong to the youngest generation in all areas.

Fissure monazite growth domain ages constrain the following episodes of fissure formation.

#### Cretaceous (eo-Alpine) episodes

**110–90 Ma**

**Fig. 6:**
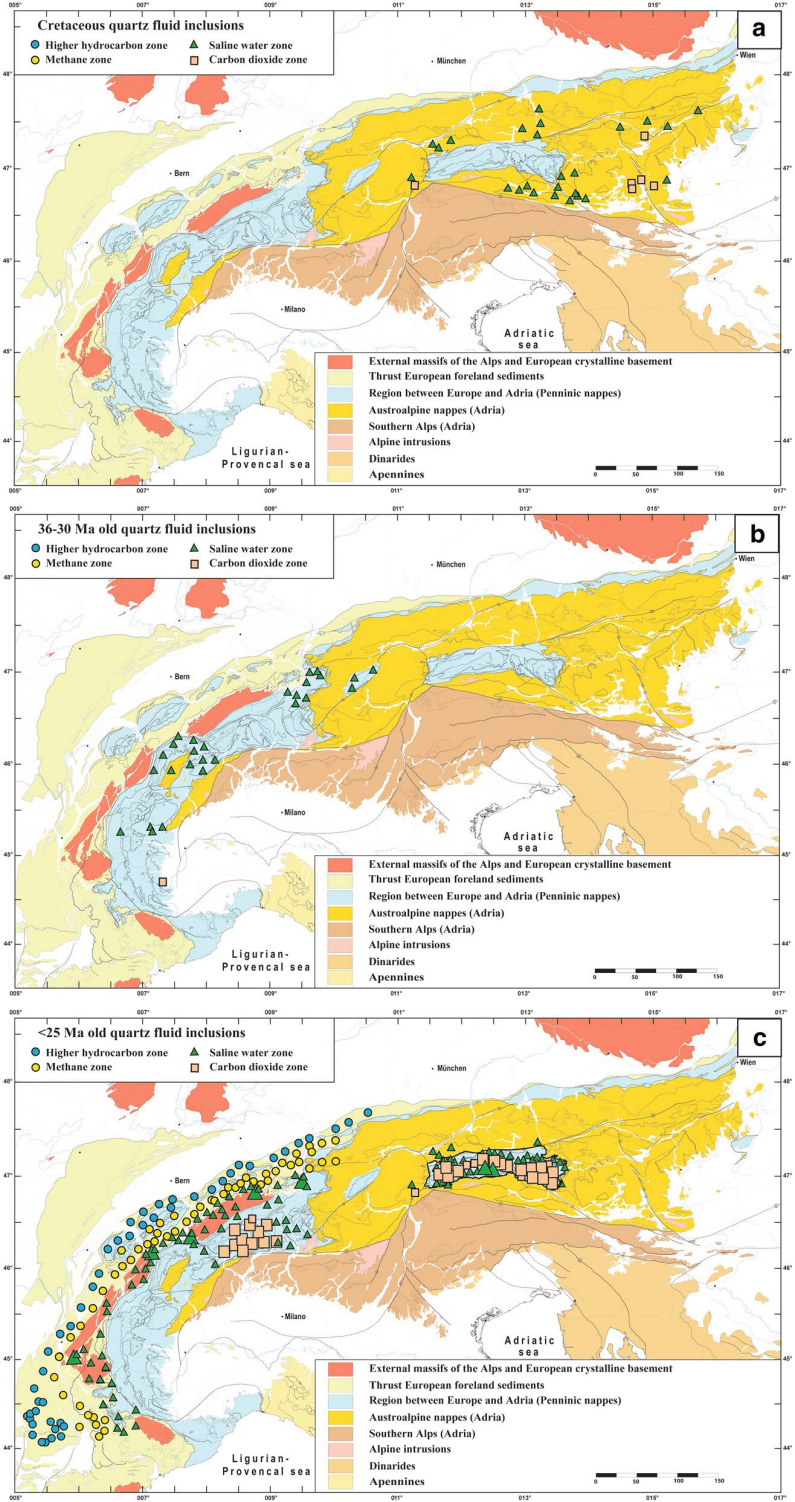
Simplified tectonic map of the Alps (based on Bousquet et al., 2012b; Schmid et al., 2004), showing the prevailing quartz fluid inclusion compositions compiled in Poty et. al. (2007) and this study, attributed to tectonic episodes constrained by fissure monazite age dating. **a** Fissures located in Austroalpine units formed during the Cretaceous (eo-Alpine) Barrow-type metamorphism and subsequent Gosau basin formation at 90–70 Ma old. **b** Areas containing 36–30 Ma old fissure monazites correlate with fluid inclusion data that formed during greenschist to amphibolite facies overprinting of high- to ultrahigh-pressure areas (Briançonnais and Piemontais zones). **c** 22–5 Ma old fissure monazites domains correlating with areas where quartz fluid inclusions became trapped during regional scale metamorphism in association with the exhumation of the Tauern metamorphic dome, the Engadine Window (and probably also the Rechnitz Window), the Lepontine metamorphic dome, and the external Aar, Gotthard, Mont-Blanc, Aiguilles Rouges, Belledonne and Pelvoux massifs. In addition, fissure formation and fluid trapping also occurred in association with strike-slip faulting in the Central and Western Tauern Window, in the Lepontine dome, in the overprinted high-pressure units of the Western Alps, and in the external massifs. This is due to escape tectonics following maximal steepening of the external massifs. 10–5 Ma old monazite growth domains indicate subsequent episodic reactivation of strike-slip movements

**Fig. 7:**
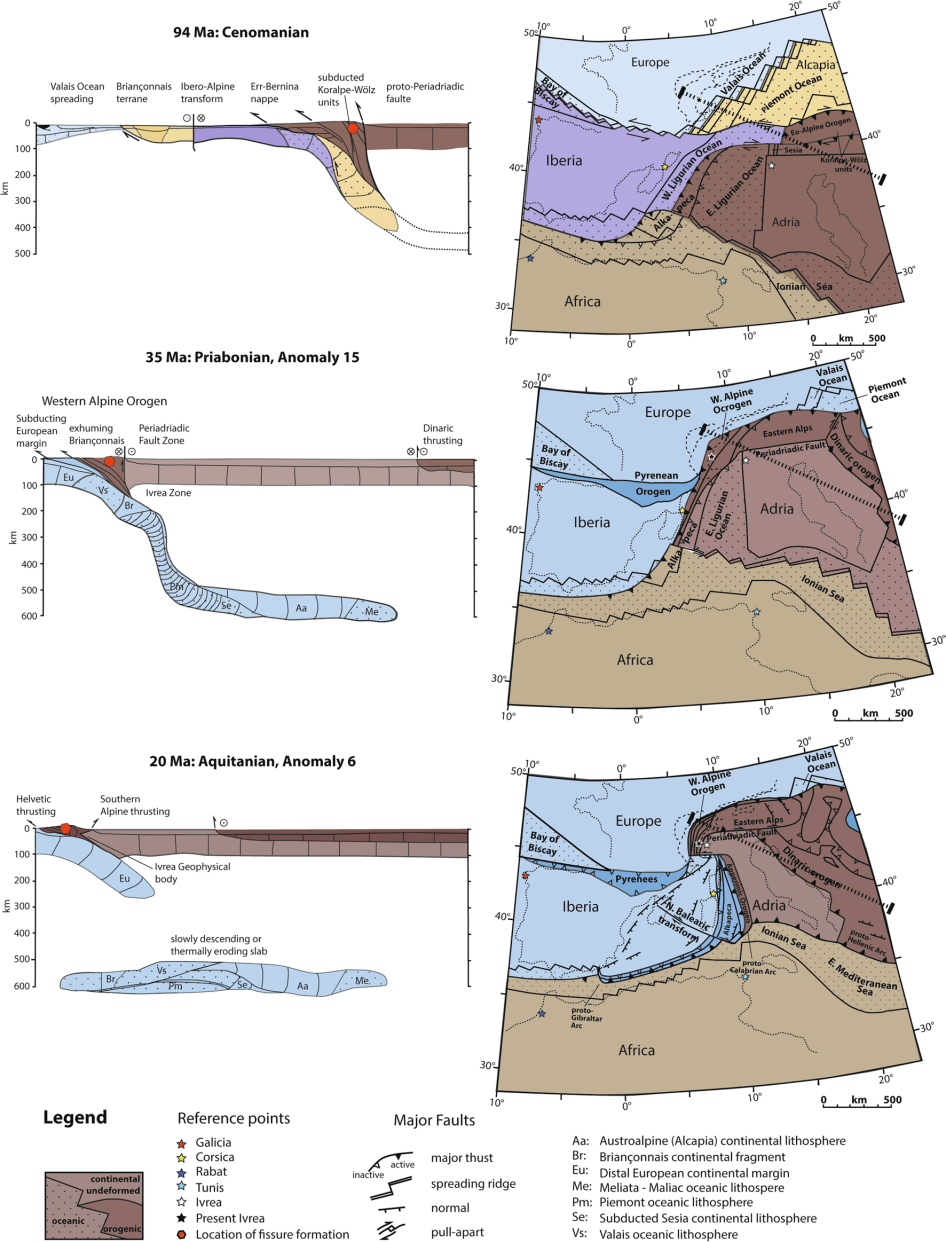
Paleogeographic reconstruction and cross sections from Handy et. al. (2010), depicting the main stages of fissure formation in association with extensional exhumation following nappe stacking during the Cretaceous and Cenozoic at 94 Ma, 35 Ma and 20 Ma. Open fissure form typically at 450–550 °C and 0.3–0.6 GPa or below (Mullis et al. 1994; Poty et al. 2007, 2018). The situation at 94 Ma is associated with exhumation of the Koralpe-Saualpe region. The situation at 35 Ma depicts the early exhumation of the high-pressure units in the future Western Alps, following the subduction of the Penninic units. Open fissures are NW striking and vertical in orientation. The reconstruction at 20 Ma shows the situation during early exhumation of the Tauern and Lepontine metamorphic and structural domes and of the external massifs. Open fissure forming until ~ 12 Ma are vertical in orientation and “N–S” oriented in the metamorphic domes (Fig. 4). Open fissure in the external massifs are horizontal in orientation (Fig. 4) and caused by reverse faulting (vertical foliation and down-dip lineation)

This time range covers the eo-Alpine (Cretaceous) oceanic and continental subduction followed by continental thickening (e.g., Schuster et al., 2004; Thöni, 1999) including HP-LT and Barrovian metamorphism (e.g., Bousquet et al., 2012b; Frey et al. 1999). Locally, fissures, which formed at the latest around ~ 90 Ma (Bergemann et al., 2018; Fig. 5) are preserved. Depending on the metamorphic grade reached, fissure fluid is dominated by CO_2_ or saline water (Fig. 6a). Fissures and clefts are vertical and ENE striking in the metamorphic regions of Saualpe-Koralpe (Koralpe-Wölz and Silvretta-Sekau Nappe Systems; e.g., Schuster et al., 2013) but very few data are available. Fissures have also been reported from the Pohorje ultra-high pressure unit (Niedermayr, 1992) and presumably formed at a similar time. A paleogeographic reconstruction from Handy et. al. (2010), displaying in which tectonic situation Cretaceous fissure formation occurred, is shown in Fig. 7.

In the region south of the eastern Tauern Window, thermochronology shows Cenozoic metamorphic overprinting (e.g., Rosenberg & Berger, 2009; Rosenberg et al., 2015, 2018). Although we cannot exclude that Cenozoic fissures may exist in this area, we attribute these fissures to have formed during the Cretaceous orogeny (Fig. 6). Unfortunately, data are also lacking for the Austroalpine regions west of the Tauern Window. However, mica cooling ages (Satir, 1975; Thöni, 1981) indicate an eo-Alpine metamorphism for this region, implying fissures in this area to be also Cretaceous in age.

**90–70 Ma**

Following exhumation, extensional movements along normal faults led to subsidence and formation of the Gosau basins in the Eastern Alps between 90 and 70 Ma (e.g., Wagreich, 1995). Locally, existing fissure monazite recrystallized during this tectonic activity (Bergemann et al., 2018). Quartz fluid inclusion data attributed to this deformation stage are shown in Fig. 6a.

#### Cenozoic episodes

**36–30 Ma**

The subduction of the European oceanic and following continental lithosphere produced the different HP-LT metamorphism in the Western Alps (e.g., Agard et al., 2003; Berger & Bousquet, 2008). Slab breakoff and underthrusting of (ultra)high-pressure metamorphic units by the European continental crust caused rapid post-nappe exhumation, decompression and metamorphic overprinting of the HP assemblages at greenschist to amphibolite facies conditions (e.g., Manzotti et al., 2018; Schenker et al., 2015). Schenker et. al. (2015) proposed that decompression of the HP Dora Maira, Monte Rosa, Gran Paradiso, Adula-Cima Lunga and Zermatt-Saas units down to ~ 1 GPa occurred within < 2 Ma. The associated fluid-assisted fissure formation may have been triggered by this decompression tectonics. The vertical to sub-vertical fissures in the HP Briançonnais and Piemontais units are all roughly NNW-striking (Fig. 4) indicating an ENE striking of σ3. Fissures of this age show a similar orientation from the Zermatt-Saas to the Dora Maira regions (Fig. 4) and are oriented perpendicular to the prevailing flat foliation and lineation. This probably indicates that the younger counterclockwise rotations of units (Collombet et al., 2002; Maffione et al., 2008; Thomas et al., 1999) related to the opening of the Ligurian basin mainly affected units located south of Dora Maira/Pelvoux regions.

**Fig. 8:**
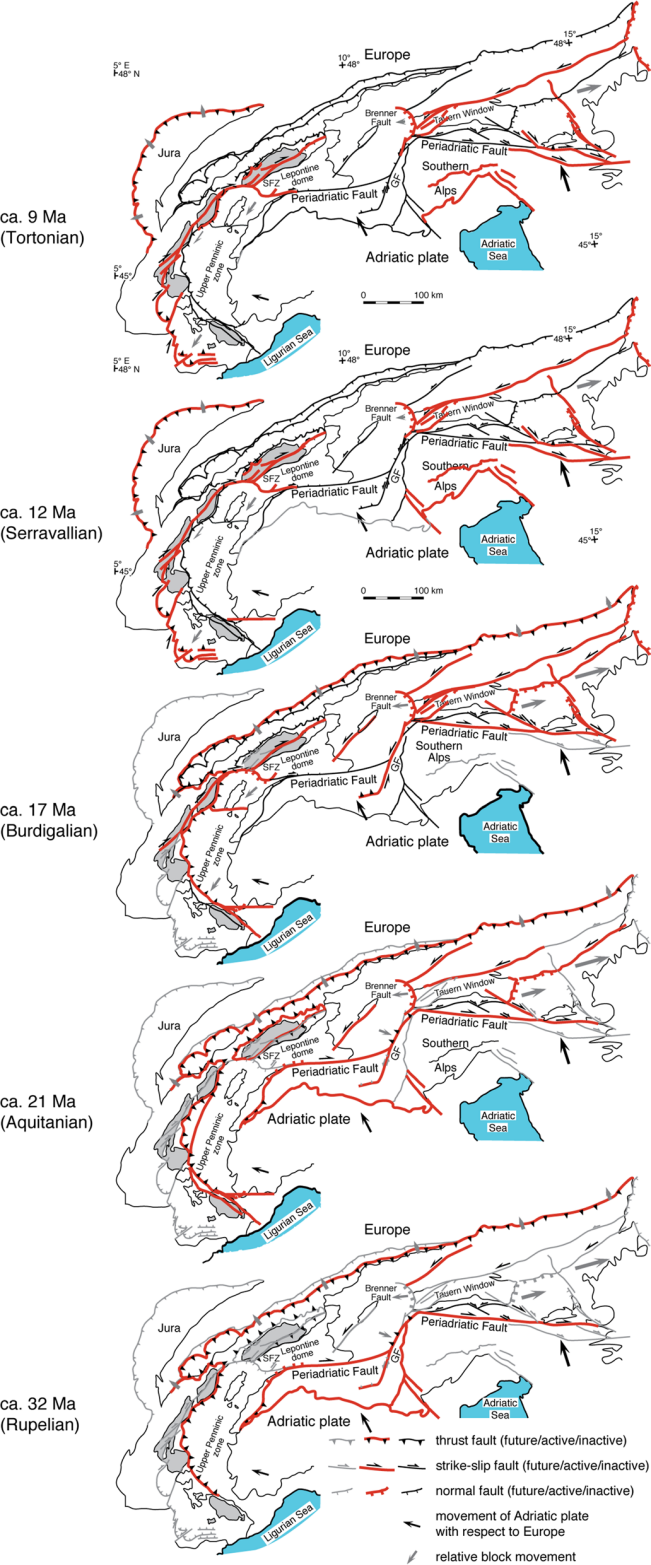
Simplified tectonic map of the Alps based on Pleuger et. al. (2012), complemented with recent data. In addition to references listed in Pleuger et. al. (2012), fissure monazite age data (Bergemann et al. 2017, 2018, 2019, 2020; Berger et al., 2013; Gasquet et al., 2010; Gnos et al., 2015; Grand’Homme et al. 2016a; Janots et al., 2012; Ricchi et al., 2019, 2020a, 2020b) and data from Schönborn (1992) were considered. Using the present tectonic constellation, the drawings show schematically Cenozoic faults active at 32 Ma, 21 Ma, 17 Ma, 12 Ma and 9 Ma. During Alpine collision, frontal faulting shifted progressively to more external parts, but fault and thrust reactivation occurred also in internal parts. The oldest monazites dated in association with strike-slip faulting derive from the exhumed high-pressure units of the Western Alps and the Argentera Massif. Fissure monazites recording < 12 Ma fault activity are mainly found in the Central and Western Tauern Window (Ricchi et al., 2020a), the northern, western and southern limits of the Lepontine dome (Bergemann et al., 2020), and in all the external massifs, except Argentera (e.g., Bergemann et al., 2019; Ricchi et al., 2020b; Fig. 4). Many of the faults active at 9 Ma are still active today

Monazite from fissures in these HP-LT regions of the Western Alps yielded crystallization ages of 36–30 Ma (Fig. 2). A paleogeographic reconstruction from Handy et. al. (2010) for 35 Ma showing the location of fissure formation in the exhuming Briançonnais units is given in Fig. 7. Faults active at 32 Ma are shown in Fig. 8. The prevailing fissure fluid is saline water, in the Dora Maira unit also CO_2_-rich (Fig. 6b). Fissure formation is also common in rodingites in ultramafic units of the Piemontais (e.g., Piccoli et al., 2007) and probably occurred roughly coevally.

Despite the lack of fissure monazite data from HP-LT regions between the Lepontine and Tauern domes (Fig. 2), radiometric and fission track data indicate that the decompression, and hence fissure formation likely occurred between 35 and 27 Ma in the Engadine Window, between 32 and 29 Ma in the Hinterrhein region (Wiederkehr et al., 2008) and between 33 and 32 Ma in the Adula Nappe (Liati & Gebauer, 2009). The areas of decompression from the HP-LT into greenschist facies (locations without a Barrovian overprint) have a different fissure record than the areas with a Barrovian overprint. However, in some areas, the “decompression greenschist” and the “Barrovian greenschist” are difficult to distinguish (Wiederkehr et al., 2008). Fissure monazites VALS and TAMB1 from the Adula and Tambo nappes analyzed by Bergemann et. al. (2020) and listed in Table 1 crystallized in association with the exhumation of the Lepontine Dome and not during the exhumation of the high-pressure rocks.

Even though fissure mineral crystallization ages are lacking fot the western boundary of the Autroalpine to the Penninic units, fissures shown in Fig. 4 are interpreted to have formed in association with exhumation of high-pressure overprinted rocks.

**25–12 Ma**

The collision of the western Adria (Ivrea indenter) with Europe led to upward movements of the central part of the Alps in combination with strike slip movements along the Insubric Line (Schmid et al., 1989; Fig. 8). In the Lepontine dome region this led to the formation of large scale-upright asymmetric antiforms, an E–W extension and doming, with fissure orientations (Fig. 4) most commonly perpendicular to the foliation and rock lineation. This tectonic evolution seems connected to the counter-clockwise rotation of Adria. Rotation has been related to the development of basins and oceanic crust west of this part of Adria (see van Hinsbergen et al., 2020) and includes the development of the Provençal basin, Ligurian basin and the Gulf of Lyon, which developed from the Oligocene to today (e.g., Dewey et al., 1989; Facenna et al., 2001; Schmid et al., 2017; van Hinsbergen et al., 2020). The here interesting stage of oceanization in the Liguro-Provençal Basin occurred between 21 and 16 Ma and was accommodated by a ~ 50° counter-clockwise rotation of Sardinia–Corsica relative to Europa (Gattacceca et al., 2007). Paleomagnetic data (Collombet et al., 2002; Maffione et al., 2008; Thomas et al., 1999) show that this is connected to the 47–117° counterclockwise rotation of blocks inside the Liguran Alps and the area of Piemonte. This tectonic of the southern part of the Western Alps is related to stage 3 of forming the Western Alpine Arc in the sense of Schmid et. al. (2017). The related fissure formation occurs in association with greenschist and sub-greenschist facies ductile to brittle, dextral shear zones (e.g., Corsini et al., 2004). Corresponding fissures in the Argentera Massif are vertical and NE oriented (Fig. 4), and monazite yielded domain ages of 20.6 ± 0.3 Ma (Grand’Homme et al., 2016a, 2016b) to 14.4 ± 0.5 Ma (Ricchi et al., 2020b; Fig. 5). Dextral faulting along another shear zone in the Argentera Massif had started at 33.6 ± 0.6 Ma (Sanchez et al., 2011), thus during exhumation of high-pressure units in the Western Alps. However, the fissure monazite record indicates that fault activity seems to have ceased at 14.4 ± 0.5 Ma (Ricchi et al., 2020b). Faults considered active at 21 and 17 Ma are shown in Fig. 8.

Using the paleomagnetic data for backrotation brings the strike-slip faults of the Argentera Massif to a N–S orientation and this would indicate that corresponding fissures were originally E–W striking.

At ~ 22 Ma steep reverse faulting and formation of horizontal fissures and clefts started in the Mont Blanc Massif (e.g., Leloup et al., 2005; Poty et al., 2018). This reverse faulting (and related fissure generation) may have started at a slightly different age in the Aiguilles Rouges, Belledonne, Pelvoux and Aar massifs and in the Gotthard nappe. During strike-slip movements in the Maritime Alps (including the Argentera) and reverse faulting in the external massifs, fissure monazite domain ages as old as 21.7 ± 0.4 Ma developed in the eastern Tauern Window (Ricchi et al., 2020a). These fissures are related to uplift of the Tauern Window in combination with strike-slip movement (e.g., Rosenberg et al., 2018). This deformation is related to the Dolomite indenter tectonics, which may have ceased at ~ 15 Ma, consistent with the fissure monazite record of the eastern Tauern Window (Ricchi et al., 2020a). The geodynamically different movement of the Dolomite indenter (eastern Adria) versus the slightly different timing and deformation in the western part of Adria is consistent with paleogeographic reconstructions proposed by van Hinsbergen et. al. (2020).

A paleogeographic reconstruction at 20 Ma (Fig. 7) from Handy et. al. (2010) displays the tectonic situation and the location where fissure formation occurred. The stress field and related fissure orientations were changing to strike-slip movements at ~ 12 Ma, which will be discussed in the next section.

**~ 12–5 Ma**

At about 12 Ma (Fig. 8) strike-slip faults formed along the northern boundary of the Lepontine dome (Bergemann et al., 2020), along the boundaries and in sub-parallel fault zones within the external massifs and its activity has been dated with monazite (Bergemann et al., 2019; Berger et al., 2013; Gasquet et al., 2010; Grand’Homme et al., 2016; Ricchi et al., 2019). The strike-slip movements are accompanied with normal faulting (i.e. Simplon Fault; Mancktelow, 1992; Fig. 8). The kinematics of large system like the Simplon and the Rhone-Simplon faults produce veins (Haertel & Herwegh, 2014). Fissure and cleft monazites record ages as young as 5 Ma (Bergemann et al., 2020; Fig. 5) related to the strike-slip movements along the lateral ramp. Comparable young ages are found in the Aar Massif (Bergemann et al., 2017; Berger et al., 2013), along the Rhone-Simplon fault in the Lepontine Dome and the Mont Blanc/Aiguilles Rouges massifs (Bergemann et al., 2019, 2020) and in the Belledonne Massif (Gasquet et al., 2010; Grand’Homme et al., 2016). Monazite growth appears to have ceased around that time, most likely due to penetrating meteoric water giving way to very low grade cleft mineral crystallization such as zeolithes and clay minerals (e.g., Mullis et al., 2001a, 2001b; Sharp et al., 2005; Weisenberger & Bucher, 2010; Weisenberger et al., 2012).

Muscovite new growth in strike-slip faults (Schneider et al., 2013) and fissure monazite domain ages (Ricchi et al., 2020a) indicate an episodic reactivation of the strike-slip faults between ~ 12 and 9 Ma in the central and western Tauern Window (Fig. 8), probably coeval with activity along the Brenner detachment fault. Fissures that formed during these different strike-slip movements show vertical orientation (Fig. 4), roughly parallel to the older fissure generation. Faults active at 12 and 9 Ma are shown in Fig. 8.

### Fissures and the σ3 orientation

By interpreting fissures as mode 1 fractures (e.g., Twiss & Moores, 2007), the measured orientations of fissures give additional information about the stress field during their formation. However, from fissure orientation only no related movement can be inferred. Therefore, the fissure data give not the same quality of information as palaeostress analysis using additionally lineations on faults or seismic data using beach-ball analysis. Moreover, the local dynamics of cleft opening may include reorientation of the local relationship of preexisting fractures and stress field. The latter can for example be recognized by sigmoidal-shaped veins or sigmoidal ending of clefts. Such differences can locally be recognized by detail field work, but the most frequent clefts are large, more or less straight openings, developing at least initially during mode 1 fracturing.

A compilation of best estimates for the σ3 axes of palaeostress orientations based on brittle structures was provided by Bertrand and Sue (2017) and is shown in Fig. 4 together with the fissure orientation. The orientation of fissures, that formed in the 12–5 Ma range in the Lepontine dome and in the external massifs, except Argentera, are generally in good agreement with the inferred σ3 directions of Bertrand and Sue (2017) (Fig. 4). These fissures are mainly related to dextral strike-slip faulting. This is also true for the sinistral strike-slip related fissures in the Tauern Window and the estimated stress field (Bertrand et al., 2015). Stress ellipsoid orientation obtained from earthquake analysis is also comparable with this late faulting (e.g., Fréchet et al., 2011). However, the Plio-Quaternary σ3 directions of Bertrand and Sue (2017) in the Western Alps are not represented in our fissure orientation dataset (Fig. 4). The inferred Plio-Quaternary σ3 directions are influenced by the exhumation and topographic evolution of the mountain belt. In contrast to missing insights in the youngest history, fissure orientations have the potential to preserve older stress fields (Fig. 4). Combining the presented fissure orientations of > 25 Ma old fissures in the Austroalpine and the high pressure units of the Western Alps, the data show that stress fields of different age may be similar in orientation.

### Fluid evolution

In most cases, the initial fissure-filling fluid is of metamorphic origin and the composition is rock-buffered (Heijboer et al., 2003a; Mangenot et al., 2021; Mullis, 1996; Mullis & De Capitani, 2000; Mullis & Tarantola, 2015; Mullis et al., 1994, 2017; Poty, 1969; Tarantola et al., 2007, 2009). This fluid composition and physical conditions (P/T) determine the habit of quartz (Fig. 3; Mullis, 1991; Mullis et al., 1994), the dominating mineral in most fissures. On the other hand, the quartz habit allows for a fluid zone assessment where fluid inclusion data are lacking. The later fluid evolution in the same region may be different. For example, a fissure may become overprinted, as has been shown by Bergemann et. al. (2017) in the Aar Massif, where a horizontal fissure that had formed during reverse faulting was later overprinted by strike slip movements. The reopening of the fissure fluid system during a change in the stress field (from reverse faulting to strike-slip faulting) caused chemical disequilibrium within the fissures. In several areas of the saline water zone in the external massifs renewed fissure deformation was associated with localized infiltration of a CO_2_-rich fluid, causing mineral reactions, fluid unmixing and sceptre quartz (often amethyst) overgrowths on previously crystallized long prismatic or Tessin habit quartz (e.g., Burgsteiner, 2004; Hossfeld, 1977; Mullis, 1991, 1996; Mullis & De Capitani, 1997; Mullis et al., 1994; Poty, 1969; Stalder et al., 1998).

More generalized, with decreasing temperature different types of fluid evolution have been observed (Mullis et al., 1994): (1) Decrease in the amount of volatiles and dissolved salt at an increase of bulk fluid inclusion density; (2) Episodes of volatile (CH_4_ or CO_2_) enrichment leading to formation of sceptre and ‘window-shaped’ quartz (amethyst overgrowth), and (3) Strong salt enrichment due to attribution of channelized fluids derived from evaporates.

At temperatures below ~ 300 °C, meteoric water may penetrate some fissure systems (Bergemann et al., 2017; Bucher & Weisenberger, 2013; Heijboer et al., 2003b, 2006; Mullis, 2011; Mullis et al., 1994, 2001a, 2001b; Sharp et al., 2005; Wolf & Mullis, 2012; Wolf et al., 2015) and dilute the fissure-filling fluid. This is commonly associated with a late growth of zeolite minerals in the fissures due to changes in fluid composition (e.g., Weisenberger & Bucher, 2010).

## Conclusion

The combination of fluid inclusion data, crystal habit observations, isotope dating and structural data illustrate the complex and long lasting process for fissure/cleft formation in the Alps. Whereas the fluid history and the related mass transfer is locally controlled and depending on the composition of the country rock, the structural setting and timing of the fissures will give insights into the evolution of the Alps. Where fluid inclusion data are lacking, the quartz habit may be used to estimate quartz formation temperature and fluid type (Fig. 3).

The concentration of open fissures and clefts is most elevated in the Tauern Window, the Lepontine Dome and the external massifs, but fissures occur locally in all parts of the Alps (Fig. 1). The unequal distribution of fissures may be related to metamorphic grade, the connected rheology, the amount and distribution of deformation, and fluid availability. Areas with high amounts of non-localized deformation have little fissure preservation potential due to ongoing overprinting (see for example veins in the southern Lepontine dome).

Our data show that fissure formation occurred in different regions of the Alps at different times:

1. In the Austroalpine region fissure mineral formation is constrained between 90 and 70 Ma (Bergemann et al., 2018), in association with metamorphic dome formation and shear zone activity during Barrow-type metamorphism overprinting high-pressure metamorphic areas. The ENE orientation of the fissures indicates a SSE–NNW directed extension during exhumation of the Koralpe-Saualpe region.
2. In the high to ultrahigh pressure region of the Western Alps (and possibly also in corresponding units of the Tauern and Engadine Windows) fissure formation is associated with folding and exhumation of subducted material (e.g., Ceriani et al., 2001; Fügenschuh & Schmid, 2003). At least in the Western Alps this exhumation followed a cool decompression path including backthrusting and normal faulting into the greenschist facies. This led to fissure monazite crystallization between 36 and 30 Ma.
3. In the Tauern Window and the Lepontine Dome, fissure monazite crystallization occurred in association with dome formation between 22 and 12 Ma. Fissure formation started at > 22 Ma in the Tauern, and somewhat later in the Lepontine dome (Fig. 6). Reverse faulting and related large fissure formations in the external massifs started also at ~ 22 Ma. Fissure formation in association with shear zone activity occurred coevally in the Argentera Massif and in the Briançonnais zone. The fissure formation in the Argentera Massif and in the Briançonnais zone are linked to dextral strike-slip movements lasting at least until 14.4 ± 0.5 Ma (Ricchi et al., 2020b).
4. Strike-slip movements starting at ~ 12 Ma and lasting until today were producing vertical fissures and clefts in different regions of the Alps (Fig. 8). Fissure formation is concentrated (or limited) to regions. Episodic reactivation of strike-slip movements of faults and connected fissures is best documented in the fissure monazite record.

Combining different methods (isotope dating, fluid inclusions, structures) open fissures provide important complementary information about the thermal-, fluid- and geodynamic evolution of the Alps and other orogens. Where fluid inclusion data are lacking, the quartz habit can be used to constrain the fluid type. Fissures are highly concentrated in areas where the brittle/ductile transition is preserved. Some areas with exhumed medium-grade amphibolite facies of higher-grade metamorphism contain either no fissures or completely overprinted fissures described as veins. In areas of missing competence contrast of the material, the brittle–ductile transition may never be reached and fissures are hence rare or completely missing.

## Supplementary Information

**Additional file 1: Table S1.** Th–U–Pb analyses of monazite by ion microprobe (SwissSIMS). Analyses resulting in unreliable dates (e.g. presence of cracks, affected by Pbc causing high uncertainty) were not considered and are written in italic. **Figure S1.** Chemical, textural, and geochronological information for fissure monazite grains originally published in Ricchi et. al. (2019). Colour-filled circles on BSE images, correspond to ion probe spot locations. The defined growth domains (A, B, C…) are indicated on BSE images with a distinct colour code (red, orange, blue …). Spot ages considered in the weighted mean age calculations are indicated by colour-filled bars whereas spot ages only considered in the age range are indicated by open bars (bar length representing the spot age plus its 2 σ uncertainty).

## Data Availability

All data used are from published papers. Unpublished material is provided in Additional file.
