# Supplementary material for: Episodes of fissure formation in the Alps: connecting quartz fluid inclusion, fissure monazite age, and fissure orientation data
Source: Swiss J Geosci. 2021 May 10;114(1):14. doi: 10.1186/s00015-021-00391-9 (PMC8550743; doi:10.1186/s00015-021-00391-9)
Supplement: Supplementary file 1 — Additional file 1: Table S1. Th–U–Pb analyses of monazite by ion microprobe (SwissSIMS). Analyses resulting in unreliable dates (e.g. presence of cracks, affected by Pbc causing high uncertainty) were not considered and are written in italic. Figure S1. Chemical, textural, and geochronological information for fissure monazite grains originally published in Ricchi et. al. (2019). Colour-filled circles on BSE images, correspond to ion probe spot locations. The defined growth domains (A, B, C…) are indicated on BSE images with a distinct colour code (red, orange, blue …). Spot ages considered in the weighted mean age calculations are indicated by colour-filled bars whereas spot ages only considered in the age range are indicated by open bars (bar length representing the spot age plus its 2 σ uncertainty). [file 15_2021_391_MOESM1_ESM.docx]

| Table S1: Th-U-Pb analyses of monazite by ion microprobe (SwissSIMS). Analyses resulting in unreliable dates (e.g. presence of cracks, affected by Pbc causing high uncertainty) were not considered and are written in italic. | | | | | | | | | | | | | | | | | | | |
| --- | --- | --- | --- | --- | --- | --- | --- | --- | --- | --- | --- | --- | --- | --- | --- | --- | --- | --- | --- |
|  |  |  |  |  |  |  |  |  |  |  |  |  |  |  |  |  |  |  |  |
|  |  |  |  |  |  |  |  |  |  |  |  | **204-corr** | | **204-corr spot ages** | | **207-corr** | | **207-corr spot ages** | |
| **Groups** | **Analysis ID** | **U (ppm)** | **Th (ppm)** | **Th/U** | **^208^Pb/^204^Pb** | **1σ (%)** | **^207^Pb/^206^Pb** | **1σ (%)** | **^208^Pb/^232^Th** | **1σ (%)** | **f208 from 207 (%)** | **^208^Pb/^232^Th** | **1σ (%)** | **^208^Pb/^232^Th Age (Ma)** | **1σ (abs.)** | **^208^Pb/^232^Th** | **1σ (%)** | **^208^Pb/^232^Th Age (Ma)** | **1σ (abs.)** |
|  |  |  |  |  |  |  |  |  |  |  |  |  |  |  |  |  |  |  |  |
|  |  |  |  |  |  |  |  |  |  |  |  |  |  |  |  |  |  |  |  |
| A | CAVR1@18 | 183 | 18180 | 99 | 635 | 9 | 0.349 | 2.1 | 0.000727 | 1.7 | 5 | 0.0006829 | 1.7 | 13.80 | 0.23 | 0.000694 | 1.7 | **14.02** | **0.24** |
|  | CAVR1@23 | 149 | 79633 | 536 | 1076 | 5 | 0.404 | 1.7 | 0.000710 | 1.6 | 1 | 0.0006845 | 1.6 | 13.83 | 0.21 | 0.000703 | 1.6 | **14.19** | **0.23** |
|  | CAVR1@24 | 169 | 82809 | 490 | 1018 | 5 | 0.410 | 1.9 | 0.000655 | 1.6 | 1 | 0.0006301 | 1.5 | 12.73 | 0.20 | 0.000647 | 1.6 | **13.08** | **0.21** |
|  | CAVR1@25 | 195 | 38191 | 195 | 842 | 7 | 0.403 | 1.8 | 0.000707 | 1.6 | 3 | 0.0006745 | 1.6 | 13.63 | 0.21 | 0.000687 | 1.6 | **13.88** | **0.22** |
| B | CAVR1@13b | 80 | 12451 | 155 | 356 | 9 | 0.540 | 2.3 | 0.000706 | 1.6 | 7 | 0.0006290 | 1.7 | 12.71 | 0.22 | 0.000653 | 1.6 | **13.19** | **0.21** |
|  | CAVR1@16 | 70 | 13657 | 196 | 416 | 9 | 0.580 | 2.1 | 0.000723 | 1.6 | 8 | 0.0006557 | 1.7 | 13.25 | 0.22 | 0.000664 | 1.6 | **13.42** | **0.22** |
|  | CAVR1@17 | 75 | 11867 | 158 | 324 | 9 | 0.532 | 2.3 | 0.000708 | 1.9 | 9 | 0.0006236 | 2.0 | 12.60 | 0.25 | 0.000648 | 1.9 | **13.09** | **0.25** |
|  | CAVR1@19 | 216 | 20897 | 97 | 953 | 11 | 0.295 | 2.4 | 0.000683 | 1.6 | 3 | 0.0006555 | 1.6 | 13.25 | 0.21 | 0.000662 | 1.6 | **13.38** | **0.22** |
|  | CAVR1@20 | 247 | 22375 | 91 | 1120 | 12 | 0.297 | 2.6 | 0.000656 | 1.6 | 3 | 0.0006334 | 1.6 | 12.80 | 0.20 | 0.000636 | 1.6 | **12.85** | **0.20** |
|  | CAVR1@21 | 249 | 23191 | 93 | 866 | 11 | 0.293 | 2.6 | 0.000639 | 1.6 | 3 | 0.0006105 | 1.6 | 12.34 | 0.20 | 0.000618 | 1.6 | **12.50** | **0.20** |
|  | CAVR1@22 | 239 | 22532 | 94 | 747 | 10 | 0.299 | 2.5 | 0.000660 | 1.6 | 3 | 0.0006258 | 1.6 | 12.64 | 0.20 | 0.000638 | 1.6 | **12.89** | **0.21** |
| C | CAVR1@11b | 128 | 15236 | 119 | 633 | 14 | 0.368 | 3.8 | 0.000531 | 1.9 | 3 | 0.0004988 | 1.9 | 10.08 | 0.20 | 0.000514 | 1.9 | **10.38** | **0.19** |
|  | CAVR1@12b | 136 | 16508 | 121 | 1462 | 19 | 0.281 | 4.1 | 0.000561 | 1.6 | 2 | 0.0005458 | 1.6 | 11.03 | 0.18 | 0.000549 | 1.6 | **11.09** | **0.18** |
|  | CAVR1@14b | 103 | 13404 | 130 | 484 | 13 | 0.525 | 3.0 | 0.000589 | 1.7 | 7 | 0.0005421 | 1.8 | 10.95 | 0.20 | 0.000549 | 1.7 | **11.10** | **0.19** |
|  | CAVR1@15b | 109 | 13611 | 124 | 465 | 12 | 0.522 | 2.9 | 0.000604 | 1.7 | 8 | 0.0005538 | 1.8 | 11.19 | 0.21 | 0.000559 | 1.7 | **11.29** | **0.19** |
|  | CAVR1@26 | 219 | 69219 | 317 | 1184 | 7 | 0.445 | 2.0 | 0.000529 | 1.8 | 2 | 0.0005120 | 1.7 | 10.35 | 0.18 | 0.000520 | 1.8 | **10.50** | **0.19** |
|  | CAVR1@27 | 341 | 48338 | 142 | 1266 | 10 | 0.337 | 2.7 | 0.000547 | 2.0 | 2 | 0.0005303 | 1.9 | 10.72 | 0.21 | 0.000538 | 2.0 | **10.86** | **0.21** |
|  |  |  |  |  |  |  |  |  |  |  |  |  |  |  |  |  |  |  |  |
| A | GAST1@01 | 71 | 5302 | 75 | 256 | 12 | 0.473 | 2.9 | 0.000543 | 2.1 | 13 | 0.0004614 | 2.6 | 9.32 | 0.24 | 0.000473 | 2.2 | **9.57** | **0.21** |
|  | GAST1@02 | 67 | 4044 | 60 | 147 | 12 | 0.515 | 3.0 | 0.000495 | 4.3 | 19 | 0.0003648 | 4.4 | 7.37 | 0.33 | 0.000401 | 4.3 | **8.10** | **0.35** |
|  | GAST1@03 | 109 | 4911 | 45 | 135 | 11 | 0.555 | 2.6 | 0.000520 | 3.7 | 26 | 0.0003782 | 4.3 | 7.64 | 0.33 | 0.000385 | 3.8 | **7.77** | **0.29** |
|  | GAST1@04 | 87 | 3926 | 45 | 167 | 15 | 0.519 | 3.5 | 0.000448 | 2.9 | 21 | 0.0003543 | 4.4 | 7.16 | 0.31 | 0.000354 | 3.0 | **7.16** | **0.21** |
|  | GAST1@05 | 80 | 6115 | 76 | 197 | 13 | 0.531 | 3.0 | 0.000461 | 2.6 | 16 | 0.0003704 | 3.2 | 7.49 | 0.24 | 0.000386 | 2.6 | **7.81** | **0.20** |
|  | GAST1@06 | 60 | 4307 | 72 | 135 | 11 | 0.607 | 2.6 | 0.000572 | 5.2 | 25 | 0.0004080 | 4.8 | 8.24 | 0.40 | 0.000430 | 5.2 | **8.68** | **0.45** |
|  | GAST1@07 | 44 | 3761 | 85 | 176 | 13 | 0.590 | 3.1 | 0.000572 | 3.5 | 19 | 0.0004543 | 4.1 | 9.18 | 0.37 | 0.000462 | 3.6 | **9.33** | **0.33** |
|  | GAST1@12 | 49 | 3488 | 72 | 190 | 13 | 0.478 | 3.5 | 0.000564 | 2.3 | 14 | 0.0004498 | 3.3 | 9.09 | 0.30 | 0.000487 | 2.4 | **9.85** | **0.23** |
|  | GAST1@17 | 35 | 5254 | 152 | 105 | 9 | 0.740 | 2.2 | 0.000696 | 2.8 | 32 | 0.0004410 | 3.6 | 8.91 | 0.32 | 0.000471 | 2.9 | **9.51** | **0.28** |
|  | GAST1@18 | 37 | 5127 | 139 | 89 | 8 | 0.759 | 2.0 | 0.000720 | 3.4 | 40 | 0.0004166 | 4.0 | 8.42 | 0.34 | 0.000428 | 3.6 | **8.66** | **0.31** |
|  | GAST1@19 | 37 | 4244 | 114 | 85 | 8 | 0.705 | 2.0 | 0.000740 | 6.1 | 43 | 0.0004025 | 5.0 | 8.13 | 0.40 | 0.000423 | 6.2 | **8.54** | **0.53** |
|  | GAST1@20 | 43 | 3448 | 80 | 87 | 9 | 0.719 | 2.3 | 0.000724 | 5.5 | 41 | 0.0004007 | 5.2 | 8.10 | 0.42 | 0.000427 | 5.7 | **8.64** | **0.49** |
|  | GAST1@21 | 54 | 4277 | 79 | 122 | 13 | 0.686 | 2.9 | 0.000546 | 4.0 | 32 | 0.0003740 | 4.8 | 7.56 | 0.37 | 0.000370 | 4.2 | **7.48** | **0.31** |
| B | GAST1@08 | 49 | 4072 | 83 | 251 | 16 | 0.498 | 3.7 | 0.000492 | 1.9 | 13 | 0.0004160 | 2.9 | 8.41 | 0.24 | 0.000429 | 1.9 | **8.66** | **0.17** |
|  | GAST1@09 | 61 | 7502 | 123 | 320 | 15 | 0.433 | 4.1 | 0.000461 | 1.7 | 7 | 0.0004055 | 2.3 | 8.19 | 0.19 | 0.000430 | 1.7 | **8.69** | **0.15** |
|  | GAST1@10 | 66 | 7596 | 114 | 392 | 17 | 0.454 | 4.2 | 0.000458 | 1.8 | 7 | 0.0004124 | 2.3 | 8.33 | 0.19 | 0.000424 | 1.8 | **8.57** | **0.16** |
|  | GAST1@11 | 74 | 6751 | 91 | 329 | 17 | 0.428 | 4.2 | 0.000453 | 2.1 | 8 | 0.0004053 | 2.8 | 8.19 | 0.23 | 0.000416 | 2.1 | **8.41** | **0.18** |
|  | GAST1@13 | 66 | 4342 | 66 | 187 | 15 | 0.512 | 3.9 | 0.000487 | 2.3 | 14 | 0.0003866 | 3.6 | 7.81 | 0.28 | 0.000417 | 2.3 | **8.43** | **0.20** |
|  | GAST1@14 | 334 | 11904 | 36 | 503 | 16 | 0.239 | 3.3 | 0.000457 | 2.0 | 6 | 0.0004221 | 2.2 | 8.53 | 0.19 | 0.000431 | 2.0 | **8.70** | **0.17** |
|  | GAST1@15 | 347 | 12138 | 35 | 513 | 16 | 0.275 | 3.1 | 0.000457 | 1.7 | 7 | 0.0004223 | 2.0 | 8.53 | 0.17 | 0.000423 | 1.7 | **8.55** | **0.14** |
|  | GAST1@16 | 370 | 12422 | 34 | 367 | 14 | 0.306 | 3.0 | 0.000461 | 1.7 | 8 | 0.0004121 | 2.1 | 8.33 | 0.17 | 0.000422 | 1.7 | **8.53** | **0.15** |
|  |  |  |  |  |  |  |  |  |  |  |  |  |  |  |  |  |  |  |  |
|  | GOTT1@01 | 375 | 19430 | 52 | 425 | 7 | 0.278 | 1.6 | 0.000712 | 2.4 | 7 | 0.0006477 | 2.3 | 13.09 | 0.30 | 0.000662 | 2.5 | **13.37** | **0.33** |
|  | GOTT1@02 | 391 | 19150 | 49 | 427 | 8 | 0.267 | 1.7 | 0.000682 | 2.4 | 7 | 0.0006217 | 2.3 | 12.56 | 0.29 | 0.000636 | 2.4 | **12.85** | **0.31** |
|  | GOTT1@03 | 374 | 19620 | 52 | 412 | 8 | 0.275 | 1.7 | 0.000702 | 2.5 | 7 | 0.0006380 | 2.3 | 12.89 | 0.30 | 0.000651 | 2.5 | **13.16** | **0.32** |
|  | GOTT1@04 | 382 | 20841 | 55 | 376 | 7 | 0.298 | 1.6 | 0.000690 | 2.4 | 8 | 0.0006193 | 2.3 | 12.51 | 0.29 | 0.000634 | 2.4 | **12.81** | **0.31** |
|  | GOTT1@05 | 354 | 21292 | 60 | 306 | 6 | 0.337 | 1.5 | 0.000694 | 2.6 | 10 | 0.0006059 | 2.4 | 12.24 | 0.30 | 0.000625 | 2.6 | **12.63** | **0.33** |
|  | GOTT1@06 | 478 | 28244 | 59 | 323 | 6 | 0.418 | 1.2 | 0.000767 | 2.4 | 11 | 0.0006753 | 2.2 | 13.65 | 0.31 | 0.000683 | 2.4 | **13.79** | **0.33** |
|  | GOTT1@07 | 496 | 26730 | 54 | 358 | 6 | 0.385 | 1.3 | 0.000745 | 2.4 | 10 | 0.0006647 | 2.3 | 13.43 | 0.30 | 0.000671 | 2.4 | **13.55** | **0.33** |
|  | GOTT1@08 | 557 | 30159 | 54 | 288 | 6 | 0.471 | 1.2 | 0.000740 | 2.6 | 14 | 0.0006406 | 2.4 | 12.94 | 0.31 | 0.000635 | 2.6 | **12.84** | **0.34** |
|  | GOTT1@12 | 982 | 9422 | 10 | 230 | 8 | 0.173 | 1.6 | 0.000725 | 2.5 | 14 | 0.0006027 | 2.5 | 12.18 | 0.30 | 0.000625 | 2.5 | **12.63** | **0.32** |
|  | GOTT1@13 | 1425 | 26938 | 19 | 521 | 7 | 0.163 | 1.4 | 0.000666 | 2.4 | 5 | 0.0006162 | 2.3 | 12.45 | 0.29 | 0.000629 | 2.4 | **12.71** | **0.31** |
|  | GOTT1@16 | 691 | 15242 | 22 | 376 | 8 | 0.202 | 1.6 | 0.000736 | 2.5 | 8 | 0.0006626 | 2.4 | 13.39 | 0.32 | 0.000679 | 2.5 | **13.73** | **0.34** |
|  | GOTT1@17 | 761 | 14455 | 19 | 507 | 10 | 0.183 | 1.8 | 0.000699 | 2.4 | 7 | 0.0006454 | 2.4 | 13.04 | 0.31 | 0.000649 | 2.4 | **13.10** | **0.32** |
|  | GOTT1@18 | 839 | 14861 | 18 | 509 | 10 | 0.174 | 1.8 | 0.000697 | 2.4 | 7 | 0.0006438 | 2.4 | 13.01 | 0.31 | 0.000649 | 2.4 | **13.12** | **0.32** |
|  | GOTT1@19 | 930 | 13822 | 15 | 373 | 9 | 0.168 | 1.8 | 0.000687 | 2.4 | 8 | 0.0006161 | 2.4 | 12.45 | 0.29 | 0.000634 | 2.4 | **12.81** | **0.31** |
|  | GOTT1@20 | 940 | 13968 | 15 | 424 | 10 | 0.163 | 1.8 | 0.000696 | 2.4 | 7 | 0.0006327 | 2.4 | 12.78 | 0.30 | 0.000645 | 2.4 | **13.02** | **0.32** |
|  | GOTT1@14 | 1500 | 27606 | 18 | 556 | 9 | 0.171 | 1.6 | 0.000637 | 2.4 | 6 | 0.0005936 | 2.3 | 12.00 | 0.28 | 0.000600 | 2.4 | **12.11** | **0.29** |
|  | GOTT1@15 | 1787 | 29700 | 17 | 531 | 10 | 0.190 | 1.7 | 0.000619 | 2.5 | 7 | 0.0005736 | 2.4 | 11.59 | 0.28 | 0.000576 | 2.5 | **11.63** | **0.29** |
|  |  |  |  |  |  |  |  |  |  |  |  |  |  |  |  |  |  |  |  |
| A | JOLI2@01 | 225 | 8604 | 38 | 144 | 9 | 0.471 | 2.1 | 0.000513 | 3.2 | 23 | 0.0003754 | 3.4 | 7.59 | 0.26 | 0.000397 | 3.3 | **8.02** | **0.26** |
|  | JOLI2@07 | 44 | 7422 | 168 | 490 | 17 | 0.304 | 3.6 | 0.000435 | 2.4 | 7 | 0.0004010 | 2.6 | 8.10 | 0.21 | 0.000405 | 2.5 | **8.19** | **0.20** |
|  | JOLI2@08 | 79 | 4486 | 57 | 257 | 15 | 0.378 | 3.4 | 0.000443 | 2.5 | 13 | 0.0003765 | 3.1 | 7.61 | 0.24 | 0.000386 | 2.5 | **7.79** | **0.20** |
|  | JOLI2@10 | 82 | 4690 | 57 | 473 | 23 | 0.243 | 5.4 | 0.000377 | 2.4 | 6 | 0.0003466 | 2.9 | 7.00 | 0.21 | 0.000356 | 2.5 | **7.20** | **0.18** |
|  | JOLI2@12 | 207 | 5221 | 25 | 101 | 7 | 0.559 | 1.7 | 0.000605 | 3.1 | 37 | 0.0003791 | 3.5 | 7.66 | 0.26 | 0.000379 | 3.2 | **7.66** | **0.25** |
|  | JOLI2@06 | 24 | 5570 | 230 | 475 | 21 | 0.210 | 4.7 | 0.000356 | 2.8 | 6 | 0.0003269 | 3.1 | 6.61 | 0.20 | 0.000334 | 2.8 | 6.75 | 0.19 |
|  | JOLI2@11 | 120 | 4409 | 37 | 329 | 23 | 0.280 | 5.3 | 0.000340 | 2.9 | 8 | 0.0003005 | 3.7 | 6.07 | 0.23 | 0.000313 | 2.9 | 6.32 | 0.19 |
| B | JOLI2@1b | 86 | 24235 | 282 | 1077 | 14 | 0.206 | 3.6 | 0.000395 | 2.5 | 2 | 0.0003809 | 2.4 | 7.70 | 0.19 | 0.000387 | 2.5 | **7.83** | **0.19** |
|  | JOLI2@03 | 90 | 17018 | 189 | 627 | 14 | 0.207 | 4.1 | 0.000366 | 2.4 | 3 | 0.0003430 | 2.4 | 6.93 | 0.17 | 0.000356 | 2.4 | **7.19** | **0.17** |
|  | JOLI2@04 | 89 | 19259 | 216 | 652 | 13 | 0.250 | 3.5 | 0.000383 | 2.5 | 3 | 0.0003602 | 2.5 | 7.28 | 0.18 | 0.000370 | 2.5 | **7.48** | **0.19** |
|  | JOLI2@05 | 89 | 13944 | 157 | 524 | 11 | 0.296 | 2.8 | 0.000417 | 2.4 | 5 | 0.0003875 | 2.4 | 7.83 | 0.19 | 0.000397 | 2.4 | **8.02** | **0.20** |
|  | JOLI2@02 | 131 | 22523 | 171 | 860 | 19 | 0.271 | 4.4 | 0.000287 | 2.5 | 3 | 0.0002739 | 2.5 | 5.54 | 0.14 | 0.000277 | 2.5 | **5.60** | **0.14** |
|  | *JOLI2@09* | *69* | *1695* | *25* | *88* | *13* | *0.572* | *2.8* | *0.000570* | *8.2* | *43* | *0.0003190* | *7.2* | *6.45* | *0.46* | *0.000323* | *8.4* | ***6.52*** | ***0.55*** |
|  |  |  |  |  |  |  |  |  |  |  |  |  |  |  |  |  |  |  |  |
| A | MUTT1@06 | 149 | 14462 | 97 | 726 | 11 | 0.275 | 2.7 | 0.000672 | 2.4 | 3 | 0.0006365 | 2.4 | 12.86 | 0.31 | 0.000651 | 2.4 | **13.16** | **0.32** |
|  | MUTT1@07 | 135 | 17356 | 128 | 575 | 8 | 0.309 | 2.5 | 0.000647 | 2.4 | 3 | 0.0006039 | 2.3 | 12.20 | 0.29 | 0.000629 | 2.4 | **12.72** | **0.31** |
|  | MUTT1@08 | 169 | 8851 | 52 | 407 | 11 | 0.269 | 2.7 | 0.000647 | 2.6 | 5 | 0.0005884 | 2.6 | 11.89 | 0.30 | 0.000614 | 2.6 | **12.40** | **0.32** |
|  | MUTT1@09 | 126 | 5134 | 41 | 230 | 10 | 0.332 | 2.7 | 0.000645 | 2.9 | 10 | 0.0005414 | 3.0 | 10.94 | 0.33 | 0.000580 | 2.9 | **11.73** | **0.35** |
|  | MUTT1@10 | 200 | 12459 | 62 | 485 | 10 | 0.275 | 2.6 | 0.000633 | 2.4 | 4 | 0.0005850 | 2.4 | 11.82 | 0.28 | 0.000605 | 2.4 | **12.23** | **0.30** |
| B | MUTT1@11 | 57 | 8475 | 148 | 468 | 13 | 0.553 | 2.7 | 0.000665 | 2.5 | 9 | 0.0006102 | 2.5 | 12.33 | 0.31 | 0.000604 | 2.5 | **12.20** | **0.31** |
|  | MUTT1@12 | 72 | 7235 | 101 | 234 | 9 | 0.561 | 2.3 | 0.000682 | 2.7 | 13 | 0.0005730 | 2.8 | 11.58 | 0.32 | 0.000592 | 2.8 | **11.97** | **0.33** |
|  | MUTT1@17 | 79 | 6073 | 77 | 621 | 16 | 0.366 | 3.3 | 0.000664 | 2.5 | 6 | 0.0006226 | 2.5 | 12.58 | 0.32 | 0.000625 | 2.5 | **12.63** | **0.31** |
|  | MUTT1@18 | 87 | 6430 | 74 | 207 | 10 | 0.520 | 2.3 | 0.000614 | 2.5 | 16 | 0.0004998 | 2.8 | 10.10 | 0.28 | 0.000516 | 2.6 | **10.42** | **0.27** |
|  | MUTT1@19 | 97 | 8079 | 83 | 253 | 11 | 0.528 | 2.5 | 0.000637 | 2.9 | 14 | 0.0005392 | 3.0 | 10.90 | 0.33 | 0.000549 | 3.0 | **11.09** | **0.33** |
|  | MUTT1@20 | 102 | 6837 | 67 | 185 | 10 | 0.563 | 2.5 | 0.000611 | 3.0 | 18 | 0.0004833 | 3.2 | 9.77 | 0.31 | 0.000504 | 3.1 | **10.18** | **0.31** |
| C | MUTT1@01 | 22 | 12350 | 561 | 1136 | 15 | 0.347 | 3.7 | 0.000625 | 2.4 | 2 | 0.0006037 | 2.4 | 12.20 | 0.29 | 0.000610 | 2.4 | **12.33** | **0.30** |
|  | MUTT1@02 | 26 | 12355 | 473 | 557 | 11 | 0.409 | 3.2 | 0.000625 | 2.4 | 3 | 0.0005813 | 2.4 | 11.75 | 0.28 | 0.000604 | 2.4 | **12.20** | **0.30** |
|  | MUTT1@03 | 24 | 12046 | 503 | 649 | 12 | 0.439 | 3.2 | 0.000624 | 2.4 | 4 | 0.0005873 | 2.4 | 11.87 | 0.29 | 0.000601 | 2.4 | **12.15** | **0.30** |
|  | MUTT1@04 | 26 | 12193 | 470 | 530 | 11 | 0.477 | 3.1 | 0.000588 | 2.5 | 4 | 0.0005474 | 2.4 | 11.06 | 0.27 | 0.000562 | 2.5 | **11.35** | **0.28** |
|  | MUTT1@05 | 26 | 12446 | 484 | 433 | 10 | 0.520 | 2.9 | 0.000619 | 2.4 | 5 | 0.0005635 | 2.4 | 11.39 | 0.27 | 0.000586 | 2.4 | **11.83** | **0.29** |
|  |  |  |  |  |  |  |  |  |  |  |  |  |  |  |  |  |  |  |  |
| A | NEAT1@01 | 49 | 30841 | 626 | 580 | 8 | 0.583 | 2.1 | 0.000597 | 2.5 | 5 | 0.0005573 | 2.4 | 11.26 | 0.27 | 0.000569 | 2.5 | **11.50** | **0.28** |
|  | NEAT1@02 | 48 | 30271 | 636 | 739 | 9 | 0.510 | 2.5 | 0.000586 | 2.4 | 3 | 0.0005564 | 2.3 | 11.24 | 0.26 | 0.000569 | 2.4 | **11.49** | **0.28** |
|  | NEAT1@03 | 54 | 31350 | 576 | 778 | 9 | 0.517 | 2.4 | 0.000580 | 2.5 | 3 | 0.0005511 | 2.4 | 11.14 | 0.27 | 0.000560 | 2.5 | **11.32** | **0.28** |
|  | NEAT1@04 | 53 | 30845 | 577 | 648 | 9 | 0.499 | 2.5 | 0.000581 | 2.4 | 3 | 0.0005462 | 2.3 | 11.04 | 0.26 | 0.000562 | 2.4 | **11.36** | **0.28** |
|  | NEAT1@05 | 52 | 31053 | 598 | 789 | 9 | 0.517 | 2.4 | 0.000585 | 2.4 | 3 | 0.0005563 | 2.3 | 11.24 | 0.26 | 0.000565 | 2.4 | **11.41** | **0.28** |
| B | NEAT1@06 | 56 | 22458 | 403 | 356 | 7 | 0.647 | 1.8 | 0.000618 | 2.6 | 9 | 0.0005512 | 2.4 | 11.14 | 0.27 | 0.000565 | 2.6 | **11.42** | **0.30** |
|  | NEAT1@07 | 58 | 27169 | 467 | 341 | 7 | 0.646 | 1.8 | 0.000539 | 2.5 | 9 | 0.0004783 | 2.4 | 9.67 | 0.23 | 0.000491 | 2.6 | **9.92** | **0.25** |
|  | NEAT1@08 | 67 | 24123 | 360 | 343 | 8 | 0.612 | 2.1 | 0.000603 | 2.6 | 8 | 0.0005354 | 2.5 | 10.82 | 0.27 | 0.000555 | 2.6 | **11.21** | **0.29** |
|  | NEAT1@09 | 91 | 26728 | 293 | 407 | 11 | 0.594 | 2.8 | 0.000474 | 2.5 | 7 | 0.0004289 | 2.5 | 8.67 | 0.21 | 0.000443 | 2.5 | **8.95** | **0.22** |
|  | NEAT1@10 | 83 | 24972 | 302 | 509 | 11 | 0.577 | 2.7 | 0.000530 | 2.5 | 6 | 0.0004918 | 2.5 | 9.94 | 0.25 | 0.000497 | 2.5 | **10.04** | **0.25** |
|  | NEAT1@26 | 21 | 15917 | 766 | 384 | 9 | 0.714 | 2.2 | 0.000628 | 2.5 | 9 | 0.0005651 | 2.4 | 11.42 | 0.27 | 0.000571 | 2.5 | **11.53** | **0.29** |
| C | NEAT1@27 | 40 | 19182 | 480 | 473 | 11 | 0.598 | 2.8 | 0.000556 | 2.5 | 6 | 0.0005110 | 2.4 | 10.32 | 0.25 | 0.000524 | 2.5 | **10.59** | **0.26** |
|  | NEAT1@28 | 24 | 15185 | 626 | 320 | 10 | 0.694 | 2.5 | 0.000594 | 2.5 | 11 | 0.0005222 | 2.5 | 10.55 | 0.27 | 0.000528 | 2.5 | **10.66** | **0.27** |
|  | NEAT1@30 | 38 | 19808 | 520 | 499 | 12 | 0.544 | 3.4 | 0.000496 | 3.7 | 5 | 0.0004595 | 3.6 | 9.28 | 0.33 | 0.000473 | 3.7 | **9.55** | **0.35** |
|  | NEAT1@29 | 53 | 26206 | 499 | 389 | 11 | 0.589 | 2.9 | 0.000414 | 2.6 | 7 | 0.0003725 | 2.5 | 7.53 | 0.19 | 0.000384 | 2.6 | **7.76** | **0.20** |
| D | NEAT1@16 | 66 | 29764 | 454 | 1048 | 10 | 0.359 | 2.8 | 0.000577 | 2.4 | 2 | 0.0005553 | 2.4 | 11.22 | 0.26 | 0.000566 | 2.4 | **11.44** | **0.28** |
|  | NEAT1@17 | 77 | 32200 | 419 | 1424 | 12 | 0.288 | 3.6 | 0.000490 | 2.5 | 1 | 0.0004765 | 2.5 | 9.63 | 0.24 | 0.000484 | 2.5 | **9.77** | **0.25** |
|  | NEAT1@18 | 106 | 38277 | 362 | 1225 | 14 | 0.318 | 4.4 | 0.000399 | 2.5 | 1 | 0.0003871 | 2.4 | 7.82 | 0.19 | 0.000393 | 2.5 | **7.95** | **0.20** |
|  | NEAT1@19 | 93 | 37542 | 404 | 1361 | 15 | 0.311 | 4.2 | 0.000412 | 2.5 | 1 | 0.0004008 | 2.5 | 8.10 | 0.20 | 0.000406 | 2.5 | **8.20** | **0.20** |
|  | NEAT1@20 | 81 | 33684 | 414 | 1123 | 13 | 0.341 | 3.8 | 0.000440 | 2.8 | 2 | 0.0004246 | 2.8 | 8.58 | 0.24 | 0.000433 | 2.8 | **8.74** | **0.25** |
|  | NEAT1@21 | 51 | 18692 | 366 | 813 | 11 | 0.447 | 3.2 | 0.000551 | 2.4 | 3 | 0.0005252 | 2.4 | 10.61 | 0.25 | 0.000537 | 2.4 | **10.86** | **0.27** |
|  | NEAT1@22 | 80 | 28527 | 355 | 605 | 12 | 0.527 | 3.3 | 0.000432 | 2.5 | 4 | 0.0004047 | 2.4 | 8.18 | 0.20 | 0.000416 | 2.5 | **8.40** | **0.21** |
|  | NEAT1@23 | 95 | 33830 | 356 | 584 | 10 | 0.569 | 2.5 | 0.000453 | 2.5 | 5 | 0.0004226 | 2.5 | 8.54 | 0.21 | 0.000429 | 2.6 | **8.67** | **0.22** |
|  | NEAT1@24 | 81 | 30337 | 376 | 724 | 11 | 0.525 | 2.7 | 0.000490 | 2.5 | 4 | 0.0004641 | 2.4 | 9.38 | 0.23 | 0.000471 | 2.5 | **9.52** | **0.24** |
|  | NEAT1@25 | 78 | 29357 | 375 | 662 | 11 | 0.523 | 2.7 | 0.000496 | 2.5 | 4 | 0.0004674 | 2.4 | 9.44 | 0.23 | 0.000475 | 2.5 | **9.60** | **0.24** |
|  | NEAT1@15 | 94 | 17240 | 183 | 982 | 23 | 0.284 | 7.3 | 0.000341 | 2.8 | 2 | 0.0003277 | 2.8 | 6.62 | 0.18 | 0.000336 | 2.8 | **6.79** | **0.19** |
|  |  |  |  |  |  |  |  |  |  |  |  |  |  |  |  |  |  |  |  |
| A | UNTE1@13 | 314 | 26724 | 85 | 162 | 4 | 0.638 | 0.9 | 0.000937 | 2.5 | 21 | 0.0007135 | 2.1 | 14.42 | 0.30 | 0.000739 | 2.5 | **14.94** | **0.37** |
|  | UNTE1@14 | 861 | 18837 | 22 | 280 | 6 | 0.257 | 1.3 | 0.000819 | 2.6 | 11 | 0.0007055 | 2.4 | 14.26 | 0.34 | 0.000727 | 2.6 | **14.69** | **0.38** |
|  | UNTE1@15 | 433 | 17835 | 41 | 106 | 4 | 0.646 | 0.9 | 0.000973 | 2.7 | 34 | 0.0006187 | 2.2 | 12.50 | 0.27 | 0.000640 | 2.7 | **12.93** | **0.35** |
|  | UNTE1@16 | 808 | 18091 | 22 | 62 | 3 | 0.699 | 0.8 | 0.001393 | 4.2 | 56 | 0.0005226 | 2.5 | 10.56 | 0.27 | 0.000616 | 4.3 | **12.45** | **0.54** |
|  | UNTE1@17 | 841 | 35011 | 42 | 66 | 2 | 0.690 | 0.7 | 0.000976 | 3.8 | 44 | 0.0004061 | 2.2 | 8.21 | 0.18 | 0.000543 | 3.8 | **10.97** | **0.42** |
| B | UNTE1@01 | 159 | 20562 | 129 | 668 | 9 | 0.368 | 2.0 | 0.000772 | 2.4 | 5 | 0.0007277 | 2.3 | 14.70 | 0.34 | 0.000737 | 2.4 | **14.89** | **0.36** |
|  | UNTE1@06 | 526 | 9192 | 17 | 179 | 8 | 0.379 | 1.5 | 0.000831 | 4.1 | 23 | 0.0006516 | 3.6 | 13.17 | 0.48 | 0.000636 | 4.1 | **12.86** | **0.53** |
|  | UNTE1@07 | 222 | 24240 | 109 | 582 | 8 | 0.405 | 1.8 | 0.000766 | 2.4 | 6 | 0.0007150 | 2.3 | 14.45 | 0.34 | 0.000722 | 2.4 | **14.58** | **0.36** |
|  | UNTE1@08 | 207 | 49592 | 239 | 1024 | 7 | 0.354 | 1.8 | 0.000774 | 2.4 | 2 | 0.0007446 | 2.3 | 15.04 | 0.35 | 0.000758 | 2.4 | **15.31** | **0.37** |
|  | UNTE1@09 | 303 | 62590 | 207 | 1154 | 8 | 0.337 | 2.1 | 0.000717 | 2.4 | 2 | 0.0006928 | 2.4 | 14.00 | 0.33 | 0.000704 | 2.4 | **14.22** | **0.34** |
|  | UNTE1@10 | 290 | 62388 | 215 | 1028 | 8 | 0.416 | 1.9 | 0.000738 | 2.4 | 3 | 0.0007098 | 2.3 | 14.34 | 0.34 | 0.000718 | 2.4 | **14.52** | **0.35** |
|  | UNTE1@11 | 228 | 57133 | 250 | 1001 | 7 | 0.437 | 1.8 | 0.000751 | 2.4 | 3 | 0.0007218 | 2.4 | 14.58 | 0.35 | 0.000730 | 2.4 | **14.75** | **0.36** |
|  | UNTE1@12 | 180 | 51795 | 287 | 139 | 3 | 0.782 | 0.7 | 0.000937 | 2.5 | 26 | 0.0006774 | 2.0 | 13.69 | 0.27 | 0.000697 | 2.5 | **14.08** | **0.35** |
|  | UNTE1@18 | 270 | 58906 | 218 | 953 | 6 | 0.410 | 1.5 | 0.000785 | 2.4 | 3 | 0.0007528 | 2.3 | 15.21 | 0.36 | 0.000763 | 2.4 | **15.41** | **0.37** |
|  | UNTE1@19 | 195 | 63729 | 326 | 834 | 6 | 0.498 | 1.5 | 0.000747 | 2.4 | 3 | 0.0007121 | 2.3 | 14.39 | 0.33 | 0.000722 | 2.4 | **14.59** | **0.35** |
|  | UNTE1@20 | 201 | 59233 | 294 | 354 | 3 | 0.673 | 0.9 | 0.000847 | 2.4 | 9 | 0.0007549 | 2.2 | 15.25 | 0.33 | 0.000771 | 2.4 | **15.58** | **0.38** |
|  | UNTE1@21 | 231 | 61154 | 264 | 887 | 6 | 0.419 | 1.7 | 0.000715 | 2.4 | 2 | 0.0006835 | 2.3 | 13.81 | 0.32 | 0.000698 | 2.4 | **14.10** | **0.34** |
|  | UNTE1@22 | 258 | 60472 | 234 | 953 | 6 | 0.382 | 1.7 | 0.000725 | 2.4 | 2 | 0.0006958 | 2.3 | 14.06 | 0.33 | 0.000710 | 2.4 | **14.34** | **0.35** |
| C | UNTE1@02 | 210 | 31329 | 149 | 421 | 6 | 0.524 | 1.5 | 0.000677 | 2.7 | 8 | 0.0006147 | 2.5 | 12.42 | 0.31 | 0.000623 | 2.7 | **12.59** | **0.34** |
|  | UNTE1@03 | 205 | 38191 | 186 | 672 | 7 | 0.412 | 1.7 | 0.000658 | 2.4 | 4 | 0.0006205 | 2.3 | 12.54 | 0.29 | 0.000634 | 2.4 | **12.81** | **0.31** |
|  | UNTE1@04 | 243 | 19941 | 82 | 498 | 10 | 0.413 | 2.3 | 0.000697 | 2.4 | 6 | 0.0006425 | 2.4 | 12.98 | 0.31 | 0.000652 | 2.4 | **13.18** | **0.32** |
|  | UNTE1@05 | 276 | 33051 | 120 | 511 | 8 | 0.459 | 2.0 | 0.000590 | 2.5 | 6 | 0.0005456 | 2.4 | 11.02 | 0.27 | 0.000557 | 2.5 | **11.25** | **0.28** |


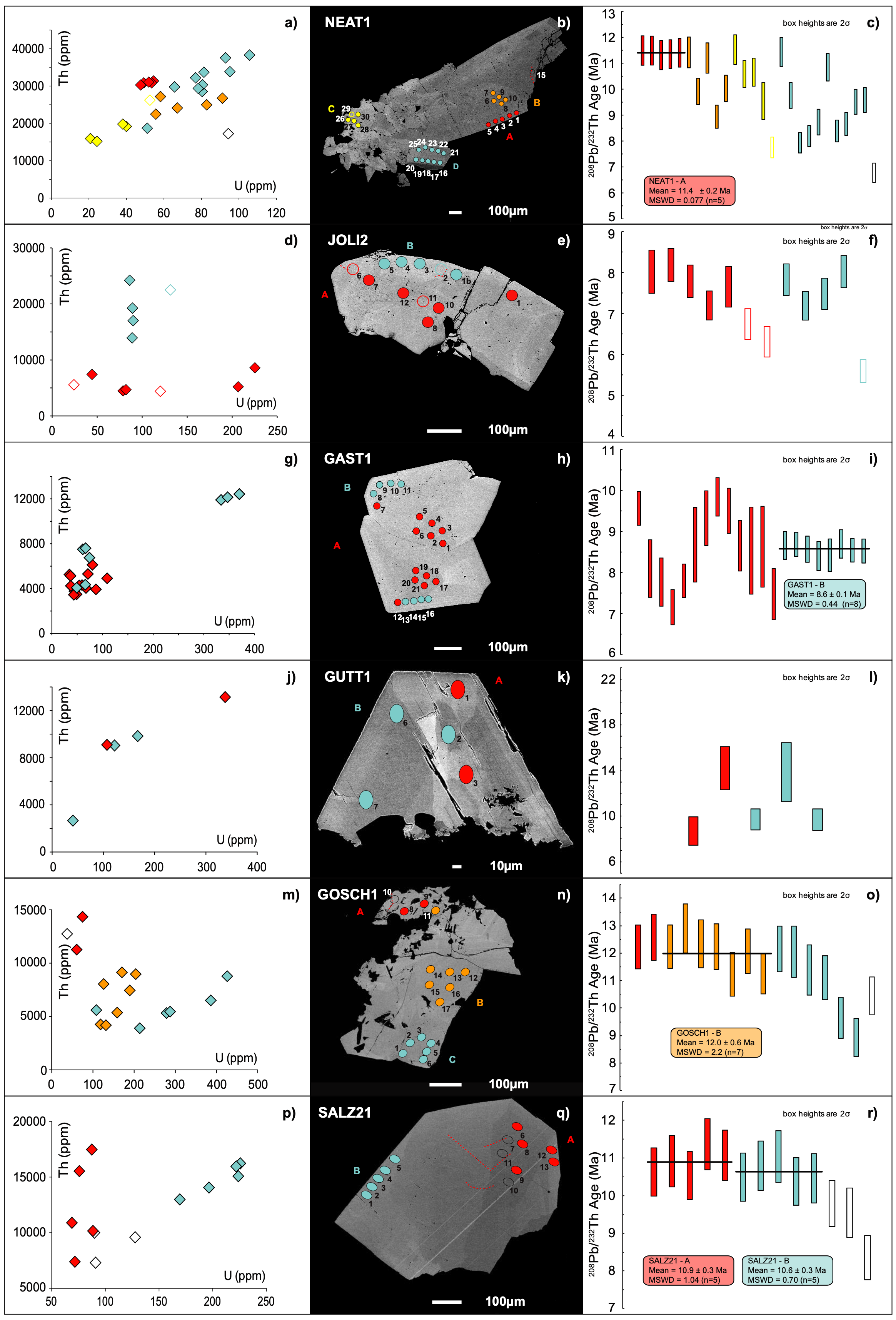

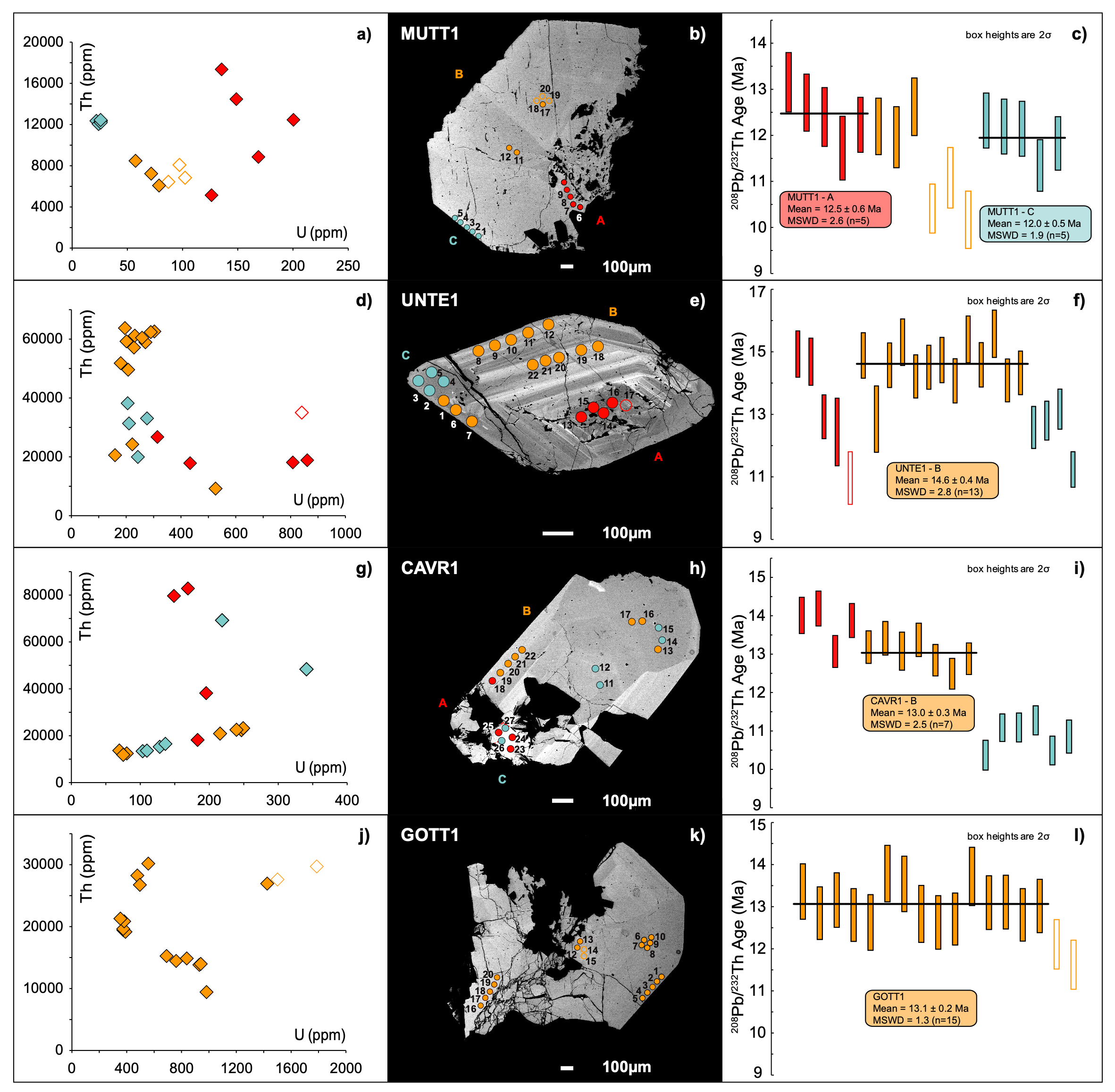


Fig. S1. Chemical, textural, and geochronological information for fissure monazite grains originally published in Ricchi et al. (2019). Colour-filled circles on BSE images, correspond to ion probe spot locations. The defined growth domains (A, B, C…) are indicated on BSE images with a distinct colour code (red, orange, blue …). Spot ages considered in the weighted mean age calculations are indicated by colour-filled bars whereas spot ages only considered in the age range are indicated by open bars (bar length representing the spot age plus its 2 σ uncertainty).

Note that fissure monazite-(Ce) ion microprobe data of grains CAVR1, GAST1, GOTT1, JOLI2, MUTT1, NEAT1 and UNTE1 from Ricchi et al., 2019 were recalculated using a different data reduction program (the Cameca customizable ion probe software (CIPS) see Ricchi et al., 2020a and b). The CIPS software is considered most appropriate for the data reduction and treatment of young fissure monazite-(Ce) crystals.
